# Autocrine IGF2 programmes β-cell plasticity under conditions of increased metabolic demand

**DOI:** 10.1038/s41598-021-87292-x

**Published:** 2021-04-08

**Authors:** Ionel Sandovici, Constanze M. Hammerle, Sam Virtue, Yurena Vivas-Garcia, Adriana Izquierdo-Lahuerta, Susan E. Ozanne, Antonio Vidal-Puig, Gema Medina-Gómez, Miguel Constância

**Affiliations:** 1 Metabolic Research Laboratories and MRC Metabolic Diseases Unit, Institute of Metabolic Science, Addenbrookes Hospital, University of Cambridge, Cambridge, CB2 0QQ UK; 2 Department of Obstetrics and Gynaecology and National Institute for Health Research, Cambridge Biomedical Research Centre, Cambridge, CB2 0SW UK; 3 Centre for Trophoblast Research, Department of Physiology, Development and Neuroscience, University of Cambridge, Cambridge, CB2 3EG UK; 4 Área de Bioquímica y Biología Molecular, Departamento de Ciencias Básicas de la Salud, Universidad Rey Juan Carlos, 28922 Alcorcón, Madrid, Spain; 5 Welcome Trust Sanger Institute, Hinxton, CB10 1SA UK; 6 Cambridge University Nanjing Centre of Technology and Innovation, Jiangbei Area, Nanjing, People’s Republic of China; 7 Present Address: Novo Nordisk A/S, 2880 Bagsværd, Denmark; 8 Present Address: Nuffield Department of Clinical Medicine, Ludwig Institute for Cancer Research, University of Oxford, Headington, Oxford, OX3 7DQ UK

**Keywords:** Metabolism, Imprinting, Developmental biology

## Abstract

When exposed to nutrient excess and insulin resistance, pancreatic β-cells undergo adaptive changes in order to maintain glucose homeostasis. The role that growth control genes, highly expressed in early pancreas development, might exert in programming β-cell plasticity in later life is a poorly studied area. The imprinted *Igf2* (insulin-like growth factor 2) gene is highly transcribed during early life and has been identified in recent genome-wide association studies as a type 2 diabetes susceptibility gene in humans. Hence, here we investigate the long-term phenotypic metabolic consequences of conditional *Igf2* deletion in pancreatic β-cells (*Igf2*^βKO^) in mice. We show that autocrine actions of IGF2 are not critical for β-cell development, or for the early post-natal wave of β-cell remodelling. Additionally, adult *Igf2*^βKO^ mice maintain glucose homeostasis when fed a chow diet. However, pregnant *Igf2*^βKO^ females become hyperglycemic and hyperinsulinemic, and their conceptuses exhibit hyperinsulinemia and placentomegalia. Insulin resistance induced by congenital leptin deficiency also renders *Igf2*^βKO^ females more hyperglycaemic compared to leptin-deficient controls. Upon high-fat diet feeding, *Igf2*^βKO^ females are less susceptible to develop insulin resistance. Based on these findings, we conclude that in female mice, autocrine actions of β-cell IGF2 during early development determine their adaptive capacity in adult life.

## Introduction

Glucose homeostasis relies on pancreatic β-cells’ ability to adapt their insulin output to meet the sensitivity of tissues such as the liver, skeletal muscle and fat to insulin. Insulin production can be augmented in two main ways: firstly, by increasing the amount produced by each β-cell, or secondly through β-cell hyperplasia (*i.e.* expanding β-cell mass via cell proliferation). Failure of these compensatory responses in the face of insulin resistance, such as during obesity, pregnancy or ageing can lead to the development of diabetes^1^. So far, several extrinsic stimuli that control the adaptive expansion of β-cell mass, as well as processes intrinsic to β-cells, which mediate their response to an increased demand for insulin, have been identified^2,3^. The intrauterine milieu, which is determined by both genetic and non-genetic factors, appears to be critical for normal β-cell development and future adaptability to metabolic stress across the lifecourse^4^. Recent genome-wide association studies (GWAS) have identified several loci linked with decreased fetal growth and increased risk for type 2 diabetes (T2D), as well as alleles associated with higher birth weight and higher T2D risk^5^. Among these, three loci are located in regions regulated by genomic imprinting, including *INS*-*IGF2*, *RB1* and *DLK1*^6^.

Insulin-like growth factor 2 (IGF2) is a major growth factor during fetal life^7^. *Igf2* is transcribed from the paternally inherited allele in most tissues^8^, and its expression in all tissues is dramatically down-regulated in mice around weaning^9^. In human, *IGF2* expression also declines with age, although significant activity is retained during adult life^9,10^. Previous studies suggested that IGF2 is required for β-cell development and function. However, much of this evidence stems from in vitro studies or analyses performed with gain-of-function rodent models, and can be summarised as follows: (1) rat β-cells overexpressing *Igf2* from a transgene were protected from interleukin-1β (IL-1β) cytokine-induced apoptosis *ex vivo*^11^ and islets isolated from neonatal rats were protected from cytokine-induced apoptosis when cultured in the presence of IGF2^12^; (2) diabetic rats transplanted with islets overexpressing *Igf2* had improved glucose tolerance compared to diabetic rats transplanted with standard islets, and β-cell replication rate was higher in *Igf2*-overexpressing islets compared to control islets after transplantation^13^; (3) Goto-Kakizaki (GK) rats that develop spontaneous diabetes had decreased IGF2 protein levels in their pancreatic bud, prior to the onset of the β-cell mass reduction^14^; (4) transgenic mice with global *Igf2* overexpression had larger pancreatic islets at the end of gestation, with increased cell replication and reduced apoptosis^15^; (5) transgenic mice with overexpression of *Igf2* in β-cells under the control of rat insulin I promoter had a threefold expansion of β-cell mass, with disrupted islet morphology, hyperglycaemia when fed a standard chow diet and overt diabetes when fed high fat diet (HFD)^16^; and (6) conditional *Igf2* deletion in pancreatic β-cells led to reduced glucose-stimulated insulin secretion (GSIS) in 24–26 weeks-old females fed chow diet, and lower GSIS in both sexes after 18 weeks of HFD feeding, associated with reduced β-cell mass in females^17^.

We recently characterised *Igf2* expression in the main pancreatic cell lineages during perinatal life^18^. We observed that at embryonic day 16 (E16) there was significant *Igf2* expression in pancreatic β-cells, which then declines rapidly after birth, being approximately two orders of magnitude lower from weaning onwards. However, we also found that the main pancreatic cell lineage expressing *Igf2* throughout perinatal life was that of mesenchyme-derived cells^18^. Accordingly, conditional *Igf2* deletion in the pancreatic mesenchyme, driven by *Nkx3.2*-Cre, led to a smaller pancreas, with reduced acinar and β-cell mass and altered glucose homeostasis when mutant females became pregnant^18^. This study highlighted an important role for pancreatic mesenchyme IGF2 in paracrine growth signalling, including the β-cell. One area that remains poorly understood is if, and how, fetal growth control genes expressed in the β-cell, such as *Igf2*, are involved in programming of diabetes susceptibility in later life. Data from Modi et al*.*^17^, and our own data on conditional *Igf2* deletions in the pancreatic endocrine lineage (*Ptf1a*-Cre)^18^, suggest that in normal physiological conditions, deletion of *Igf2* from β-cells or its precursors, have little effect on β-cell biology. However, several questions remain to be elucidated: (1) Is β-cell remodelling, which occurs in the early postnatal period^19^, affected by the embryonic deletion of β-cell *Igf2*? (2) Is β-cell mass expansion^20^ and/or glucose homeostasis impaired in pregnant mothers that lack *Igf2* in their β-cells? If yes, does it have consequences for the growth and metabolism of their offspring? (3) Does congenital obesity^21^ or HFD-induced obesity^22^, together with embryonic deletion of β-cell *Igf2* result in early programming of diabetes? By addressing these questions, we therefore aim at testing the possibility that autocrine actions of IGF2 in pancreatic β-cells during early life may program their ability to adapt to physiological challenges or conditions that cause insulin resistance throughout the life-course.

## Results

### Autocrine IGF2 is not required for early postnatal β-cell remodelling and function

**Figure 1:**
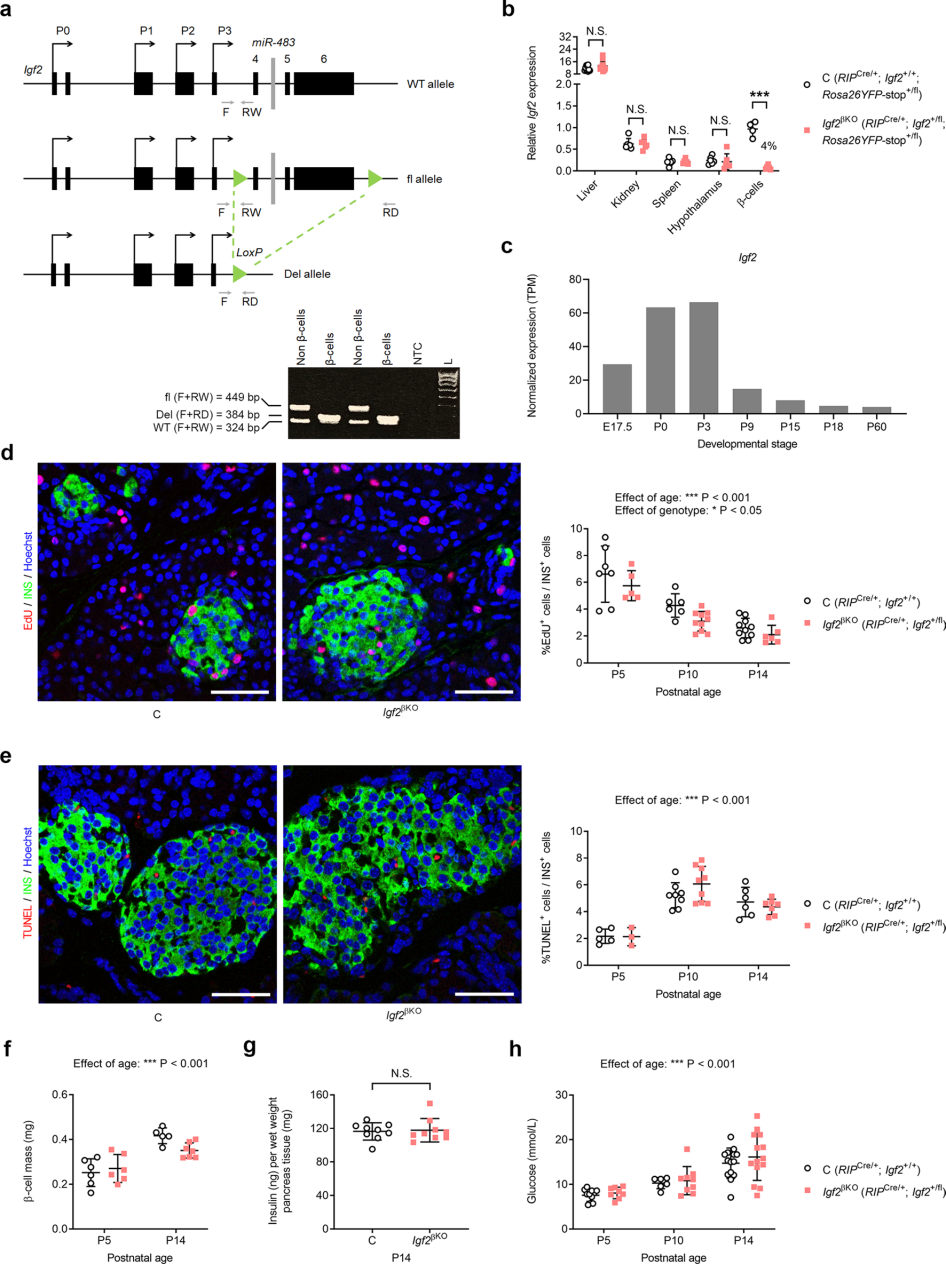
Impact of *Igf2*^βKO^ on β-cell remodelling and glucose homeostasis in early postnatal life. (**a**) Schematic representation of the *Igf2* wild-type allele (top) floxed allele (middle) and Cre-mediated deletion of the region flanked by LoxP sites (bottom) and PCR genotyping strategy showing complete deletion (Del) in pancreatic β-cells isolated by FACS, but no evidence for deletion in non β-cells (NTC—no template control, L—100 bp DNA ladder). See also the uncropped gel picture in Supplemental Fig. S1e. (**b**) Assessment of *Igf2* deletion efficiency and specificity by qRT-PCR at postnatal day 2 (P2). Data was normalised to *Ppia*, *Gapdh* and *Sdha*, used as internal controls (n = 4—12 samples/group). Residual *Igf2* mRNA levels in β-cells isolated from *Igf2*^βKO^ compared to controls is shown as %. (**c**) RNA-seq analysis of *Igf2* expression levels (TPM—transcripts per million) in pancreatic β-cells isolated by FACS (n ≥ 2 at each developmental stage), showing a rapid decline at mRNA level in post-natal life (data derived from Qiu et al*.*^26^). (**d**) Left: representative examples of P10 pancreatic sections stained for EdU (red—marker of cell proliferation), insulin (INS, green—staining β-cells) and Hoescht (blue—staining nuclei). Right: quantification of proliferating β-cells (EdU^+^/INS^+^) (n = 5–10 samples/group). (**e**) Left: representative examples of P10 pancreatic sections stained for TUNEL (red—marker of cell apoptosis), insulin (INS, green—staining β-cells) and Hoescht (blue—staining nuclei). Right: quantification of apoptotic β-cells (TUNEL^+^/INS^+^) (n = 3–9 samples/group). (**f**) Measurement of pancreatic β-cell mass using stereology at P5 and P14 (n = 5–7 samples/group). (**g**) Measurement of total pancreatic insulin content at P14, adjusted for total protein content (n = 9 samples/genotype). (**h**) Non-fasting glucose levels in peripheral blood at P5, P10 and P14 (n = 6–14 samples/group). Scale bars in (**d**) and (**e**) are 50 µm. For all graphs, data is shown as individual values, with averages ± S.D. (standard deviation). *P* values shown above the graphs correspond to *t* tests with Holm-Sidak correction for multiple testing in (**b**), two-way ANOVA tests in (**d**), (**e**), (**f**) and (**h**) and a Mann–Whitney test in (**g**). *NS* non-significant.

We achieved conditional deletion of the paternal *Igf2* allele in pancreatic β-cells (*Igf2*^βKO^) (Fig. 1a) by crossing heterozygous *Igf2* floxed male mice (*Igf2*^+/fl^)^18^ with homozygous *RIP*-Cre female mice that carry a Cre transgene under the control of the rat *Ins2* (insulin 2) promoter^23^. We used a recombinase-inducible YFP reporter under the control of the *Rosa26* locus (*Rosa26YFP*-stop^fl/fl^)^24^, which enabled us to isolate pancreatic β-cells using Fluorescence Activated Cell Sorting (FACS) to verify for efficiency and specificity of *Igf2* deletion. (Supplemental Fig. S1a–e). Paternal *Igf2*^βKO^ resulted in ~ 96% reduction of its mRNA levels in pancreatic β-cells isolated by FACS at postnatal day 2 (P2) (Fig. 1b). In contrast, *Igf2* mRNA levels were similar between *Igf2*^βKO^ and littermate controls in several other tissues tested, including in the hypothalamus (Fig. 1b), in which we observed only very few isolated YFP + cells (Supplemental Fig. 1f), in agreement with previous studies^25^.

Analysis of recently published transcriptomes of bulk β-cells isolated by FACS at several developmental time points, spanning from E17.5 to P60^26^, showed that *Igf2* mRNA levels are relatively much higher in perinatal life and decline rapidly few days after birth (Fig. 1c), in line with our own previous results^18^. To assess whether *Igf2*^βKO^ alters the dynamics of the early postnatal wave of β-cell remodelling, we first studied rates of cell proliferation at P5, P10 and P14 after intra-peritoneal administration of EdU (5-ethynyl-2′-deoxyuridine)^27^ for 6 h. The percentage of proliferating β-cells (EdU^+^/INS^+^) was significantly affected by age, decreasing from ~ 6.6% at P5 to ~ 2.6% at P14 in controls (Fig. 1d). We observed a small genotype-dependent reduction of β-cell proliferation rate across the three ages studied (Fig. 1d). Rates of β-cell apoptosis (TUNEL^+^/INS^+^) peaked around P10 (Fig. 1e), as previously reported^19^. However, there was no genotype-dependent difference in the frequency of apoptotic β-cells (Fig. 1e). Additional parameters measured in early postnatal life, including β-cell mass (Fig. 1f), total pancreatic insulin content (Fig. 1g) and non-fasting blood glucose levels (Fig. 1h) were similar between *Igf2*^βKO^ mutants and littermate controls. Altogether, our results show that the autocrine actions of IGF2 are not required for β-cell remodelling or function in early postnatal life.

### ***Igf2***^βKO^ does not alter glucose homeostasis in adult mice fed standard chow diet

**Figure 2:**
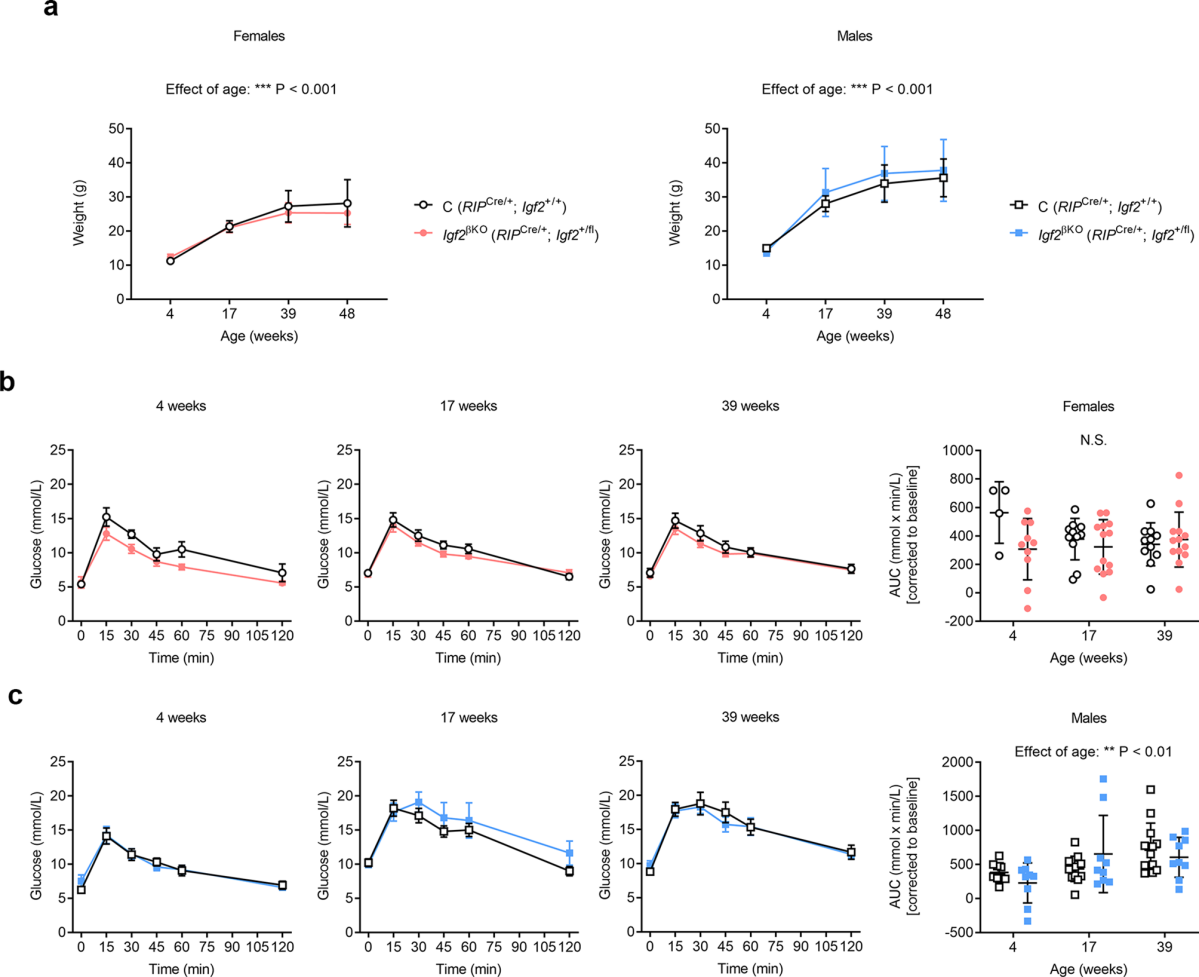
Impact of *Igf2*^βKO^ on body growth and glucose homeostasis in adult mice fed a standard chow diet. (**a**) Growth kinetics (n = 4–13 females/genotype and n = 8–13 males/genotype). Glucose tolerance tests with glucose administered by intra-peritoneal injections (ipGTTs) after over-night fasting performed in females (**b**) and males (**c**). First three panels show changes in blood glucose concentrations (y-axis), from basal pre-treatment values, with time (x-axis), after glucose administration. The graphs on the far right in (**b**) and (**c**) show area under curve (AUC) calculated during ipGTTs using the trapezoid rule and normalised to basal glucose levels (n = 4–13 females/genotype and n = 8–13 males/genotype). Data is presented as averages ± SD [panel (**a**)] or SEM [first three panels in (**b**) and (**c**)] or individual values with averages ± SD [fourth panel in (**b**) and (**c**)]. *P* values shown above graphs were calculated by mixed-effects model tests [panel (**a**)] or two-way ANOVA tests [panels (**b**) and (**c**)]. *NS* non-significant.

In order to estimate the impact of *Igf2*^βKO^ on body growth and glucose homeostasis in adult life, we followed up a cohort of mutant mice and littermate controls fed with a regular chow diet from weaning up to the age of 48 weeks. Both sexes displayed similar age-related weight gain, without any significant genotype-related difference (Fig. 2a). We monitored the impact on glucose homeostasis in both sexes by performing intra-peritoneal glucose tolerance tests (ipGTTs) after an overnight fast at the ages of 4, 17 and 39 weeks (Fig. 2b,c). Within the age range of our study, we observed significant age-related glucose intolerance only in males, as assessed by area under curve (AUC) analyses (Fig. 2c). However, neither sex had significant differences in AUC related to genotype (Fig. 2b,c). We also performed standard serum biochemistry profiling at the end of the study (*i.e.*, at the age of 48 weeks) after overnight fasting. The lipid profile showed sex-related differences, with levels of triglycerides, free fatty acids and cholesterol being significantly higher in males than in females but levels of these lipids were similar between the two genotypes in both sexes (Supplemental Fig. 2a). Corticosterone levels were lower in males than in females, but similar between mutants and controls (Supplemental Fig. 2b). Additionally, there was no significant sex-related or genotype-related differences in glucose and insulin levels after an overnight fast (Supplemental Fig. 2c). Therefore, we conclude that ablation of *Igf2* in pancreatic β-cells does not alter glucose or insulin homeostasis during adult life in mice fed a regular chow diet.

### Pregnant ***Igf2***^βKO^ females have altered glucose homeostasis, with an impact on fetal development

**Figure 3:**
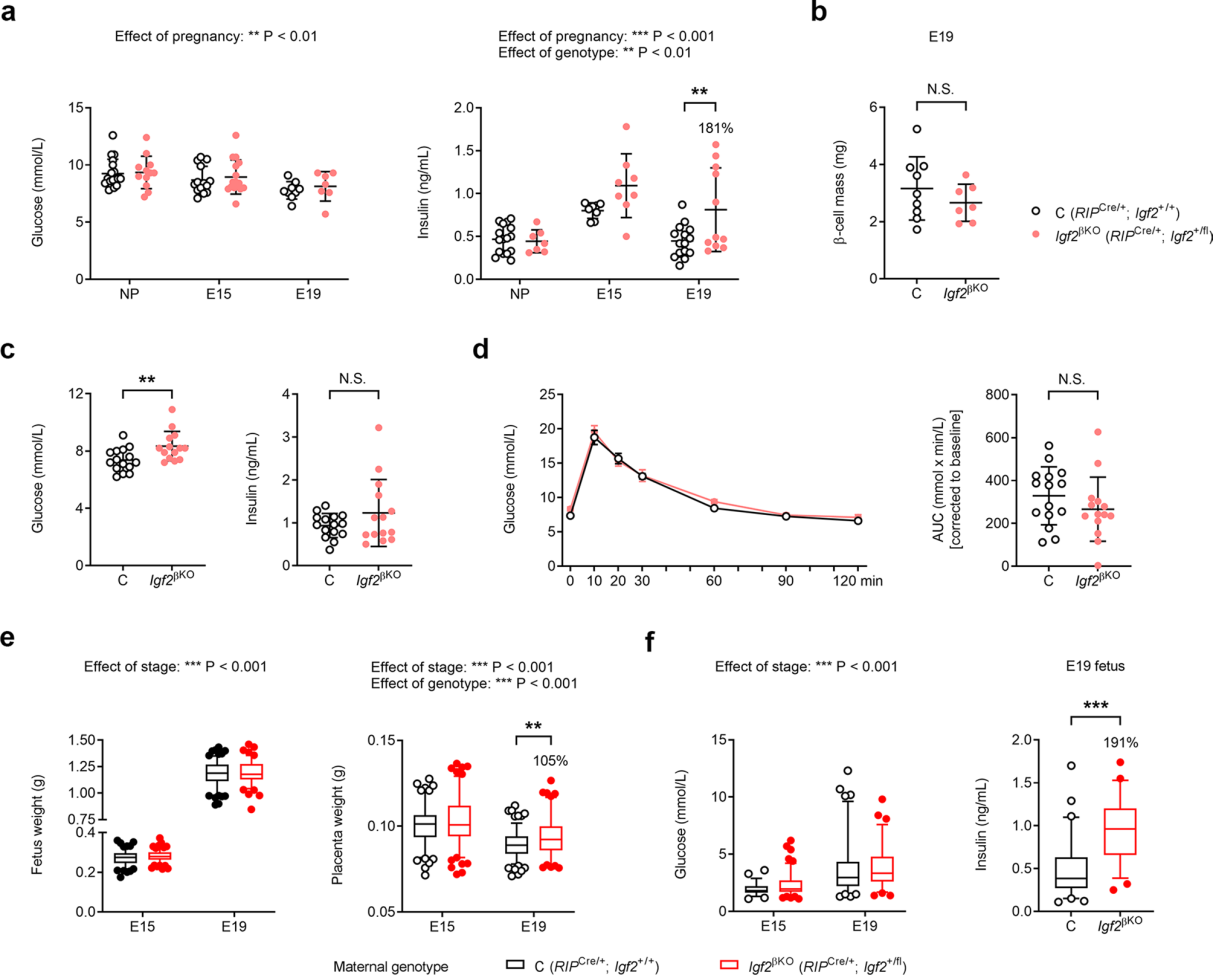
Impact of *Igf2*^βKO^ on glucose homeostasis in pregnant females and their developing conceptuses. (**a**) Non-fasting glucose and insulin levels measured in peripheral blood of 8–10 week old pregnant (E15 and E19) and non-pregnant (NP) females (n = 7–18/group). (**b**) Measurement of pancreatic β-cell mass by stereology in E19 pregnant females (n = 9 controls and n = 7 mutants). (**c**) Glucose and insulin levels measured after six hours of fasting in pregnant females (E15) (n = 15 controls and n = 14 mutants). (**d**) Left: oral glucose tolerance test (OGTT) performed in 8–10 week old pregnant females (E15) after 6 h of fasting. Changes in blood glucose concentrations (y-axis), from basal pre-treatment values, with time (x-axis), after glucose administration are shown. Right: AUC calculated using the trapezoid rule, normalised to basal glucose levels (n = 15 controls and n = 14 mutants). (**e**) Fetal and placental weights at E15 and E19 (n = 108–156 conceptuses/group). (**f**) Glucose and insulin levels in fetal blood (E19) measured after decapitation (n = 64 fetuses of control mothers and n = 54 fetuses of mutant mothers). Data is presented as individual values with averages ± SD [panels (**a**)–(**c**) and (**d**)—right side graph], averages ± SEM [panel (**d**)—left side graph], or box plots (25–75 percentiles), with whiskers extending down to the 5^th^ percentile and up to the 95^th^ percentile, and points below and above the whiskers drawn as individual points [panels (**e**) and (**f**)]. *P* values shown above the graphs were calculated by two-way ANOVA tests [panels (**a**), (**e**) and (**f**)—left side graph], unpaired *t* test with Welch’s correction [panel (**b**)], Mann–Whitney tests [panels (**c**), (**d**)—right side graph, and (**f**)]. ** correspond to p < 0.01 calculated by Sidak’s multiple comparison tests following two-way ANOVAs [panels (**a**) and (**e**)—right side graphs]. *NS* non-significant.

After establishing that the autocrine actions of pancreatic β-cell IGF2 are not required for the normal function of these cells, from in utero development up until 39 weeks of age under physiological conditions, the next goal was to find out if pancreatic β-cell IGF2 was necessary for β-cell plasticity during pregnancy, when demands for insulin increase^20^. To study the impact of *Igf2*^βKO^ on maternal adaptation to pregnancy, 6-to-8 week old *Igf2*^βKO^ and control females were timed-mated with wild-type males and analyses were performed on embryonic days E15 (*i.e.* at the peak of pregnancy-associated insulin resistance^20^) and E19 (near term). Total body weight gain during pregnancy, litter sizes and pancreas weights were indistinguishable between the two genotypes (Supplemental Fig. 3a–c). Non-fasting glucose levels decreased as gestation progressed, but remained comparable between the two genotypes (Fig. 3a). However, while in control females non-fasting circulating insulin levels increased at E15 and then returned to levels similar to non-pregnant females by E19, insulin levels remained elevated in mutant females during late gestation, being significantly higher at E19 compared to control females (Fig. 3a). Hyperinsulinemia in pregnant *Igf2*^βKO^ females was not associated with increased pancreatic β-cell mass, which was similar to controls at E19 (Fig. 3b). We did not find evidence of impaired insulin sensitivity in periphery driving the observed hyperinsulinemia at E19, with levels of well-established metabolic biochemical markers such as leptin, adiponectin and resistin^28^ being similar between the two genotypes (Supplemental Fig. 3d). The expected pregnancy-associated patterns (*i.e.* increased levels of leptin and resistin and decreased levels of adiponectin compared to non-pregnant control females) were observed (Supplemental Fig. 3d). At E15, after six hours of fasting, the pregnant *Igf2*^βKO^ mouse exhibited hyperglycaemia compared to pregnant controls, while fasting insulin levels remained comparable between the two genotypes (Fig. 3c). However, oral glucose tolerance tests (OGTTs) did not show significant differences between the two genotypes at E15 (Fig. 3d).

Next, we assessed whether maternal hyperglycaemia and hyperinsulinemia observed in *Igf2*^βKO^ females in late pregnancy had any impact on the growth and glucose metabolism of their conceptuses. Fetal weights were similar at both E15 and E19 (Fig. 3e). However, at E19, placental weights in *Igf2*^βKO^ pregnancies showed a small but statistically significant increase (5%) compared to those in control pregnancies (Fig. 3e). Levels of glucose in the fetal blood were indistinguishable in litters of *Igf2*^βKO^ versus control mothers at both E15 and E19 (Fig. 3f). However, levels of insulin measured at E19 in individual fetuses were almost double in litters of *Igf2*^βKO^ mothers (Fig. 3e). Overall, our results show that *Igf2*^βKO^ females have altered glucose homeostasis during pregnancy, associated with increased maternal and fetal insulinemia and larger placentae near term.

### ***Igf2***^βKO^ females are less susceptible to develop HFD-induced insulin resistance in adulthood

**Figure 4:**
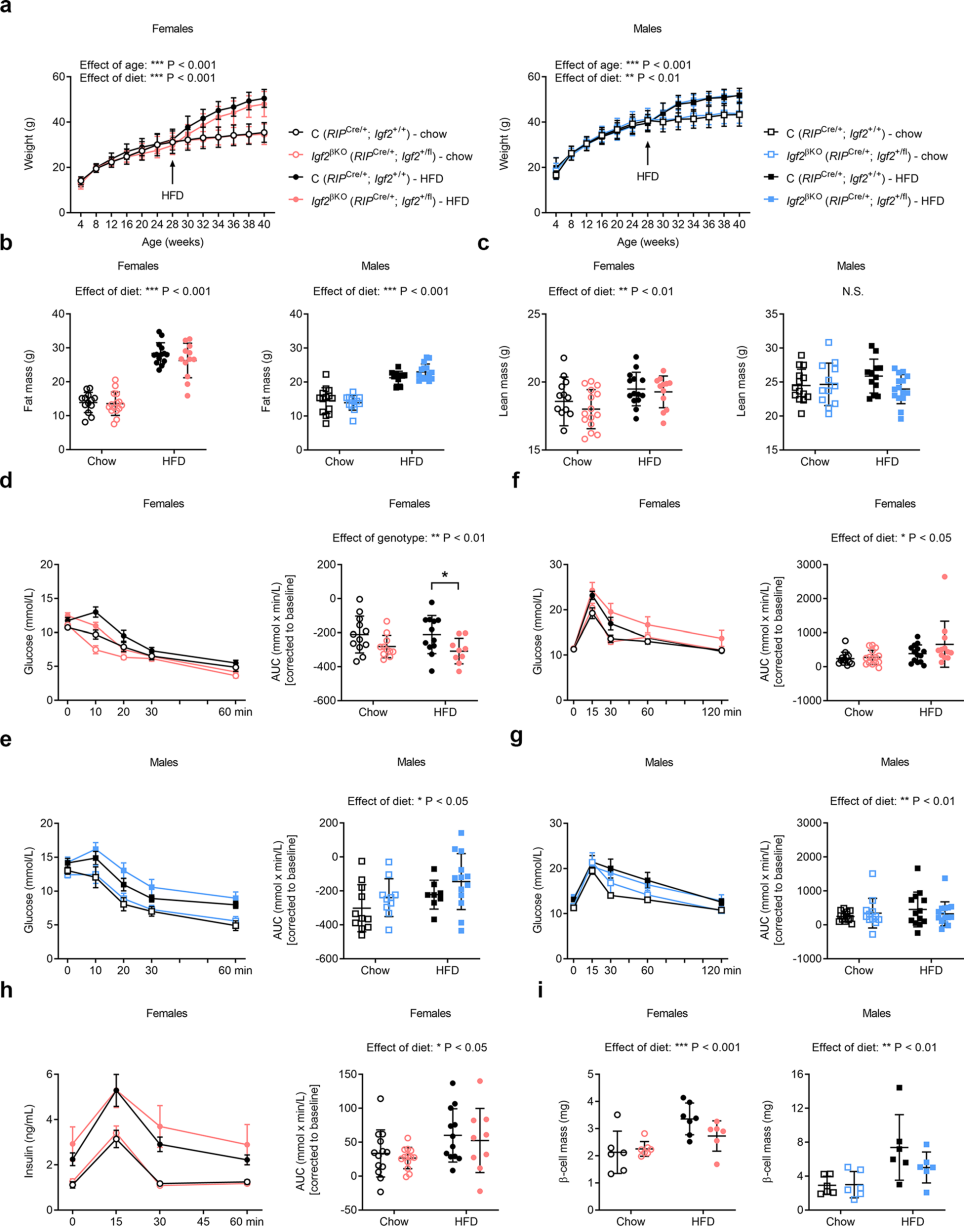
Impact of *Igf2*^βKO^ on body weight, body composition and glucose homeostasis in mice fed a HFD. (**a**) Growth kinetics in female and male cohorts (n = 12–15 mice/group) (**b**) Absolute fat mass content measured by TD-NMR in 40 week old female and male mice (n = 12–15 mice/group). (**c**) Absolute lean mass content measured by TD-NMR in 40 week old female and male mice (n = 12–15 mice/group). (**d**) and (**e**) Left: insulin tolerance tests (ITTs) performed in 40 week old females (**d**) or males (**e**) after 6 h fasting. The graphs show changes in blood glucose concentrations (y-axis), from basal pre-treatment values, with time (x-axis), after insulin administration. Right: area under curve (AUC), calculated during ITT using the trapezoid rule, normalised to basal glucose levels (n = 9–12 mice/group). (**f**) and (**g**) Left: OGTTs performed in 41 week old females (**f**) or males (**g**) after 6 h fasting. The graph shows changes in blood glucose concentrations (y-axis), from basal pre-treatment values, with time (x-axis), after glucose administration. Right: AUC, calculated during OGTT using the trapezoid rule, normalised to basal glucose levels (n = 12–15 mice/group). (**h**) Left: GSIS in 41 week old females. Right: AUC, calculated using the trapezoid rule, normalised to basal insulin levels (n = 9–13 females/group). (**i**) Measurement of pancreatic β-cell mass by stereology in 42 week old mice (n = 6–7 mice/group). Data is presented as averages ± SD (**a**), averages ± SEM [left-side graphs in panels (**d**)–(**h**)], or individual values with averages ± SD [panels (**b**), (**c**), right-side graphs in panels (**d**)–(**h**) and panel (**i**)]. *P* values shown above graphs were calculated by mixed-effects model tests [panels (**a**)], or two-way ANOVA [panels (**b**), (**c**), right-side graphs in panels (**d**)–(**h**) and panel (**i**)], * corresponds to p < 0.05 calculated by Sidak’s multiple comparison tests following two-way ANOVAs [panel (**d**)]. *NS* non-significant.

We next investigated whether HFD feeding can elicit altered glucose homeostasis in *Igf2*^βKO^ mice. To this aim, we fed a cohort of *Igf2*^βKO^ and control littermate mice with a standard chow diet from weaning (3 weeks) until the age of 28 weeks, after which half of the mice were fed HFD (60% kcal from fat) for the following 13 weeks, while the remaining half were kept on the control chow diet. Measurements of body composition by TD-NMR at the ages of 8 and 28 weeks revealed significant age-related accrual in both fat and lean mass, without any significant genotype-related effects in either of the two sexes (Supplemental Fig. 4a,b). Between the ages of 28 and 40 weeks, both sexes gained significantly more weight when fed HFD (Fig. 4a). TD-NMR measurements at the age of 40 weeks showed a significant increase in fat mass in the mice fed HFD for both sexes (Fig. 4b). Lean mass was significantly increased by HFD only in females (Fig. 4c). Neither fat mass, nor lean mass showed any significant differences related to the genotype (Fig. 4b,c). At the age of 40 weeks (i.e. 12 weeks after the HFD was introduced to half of the animals), all mice were subjected to insulin tolerance tests (ITTs), followed a week apart by OGTTs. ITTs performed in females did not show any significant diet-related differences. However, there was a significant impact of the genotype, *Igf2*^βKO^ females being less susceptible to develop insulin resistance than their littermate controls, as indicated by AUC analyses after correction to basal levels (Fig. 4d). On the contrary, ITTs performed in males revealed increased diet-associated insulin resistance, without any significant impact of the genotype (Fig. 4e). OGTTs showed that both sexes displayed a small but significant glucose intolerance induced by HFD, without any impact of the genotype (Fig. 4f,g). To investigate further the improved insulin sensitivity observed in *Igf2*^βKO^ females, we measured GSIS. Insulin levels were higher in females fed HFD at baseline and increased significantly more during OGTT (AUC *P* diet = 0.0142), without any significant impact of the genotype (Fig. 4h). β-cell mass, measured at the end of the experiment, was significantly higher in mice fed HFD for both sexes, without any difference related to the genotype (Fig. 4i). Overall, these data indicate that, when fed HFD in adulthood, *Igf2*^βKO^ mice retain the ability to adapt by expanding β-cell mass, and that *Igf2*^βKO^ females are more insulin sensitive than their littermate controls.

### ***Igf2***^βKO^ leads to exacerbated hyperglycaemia in females with congenital leptin deficiency

**Figure 5:**
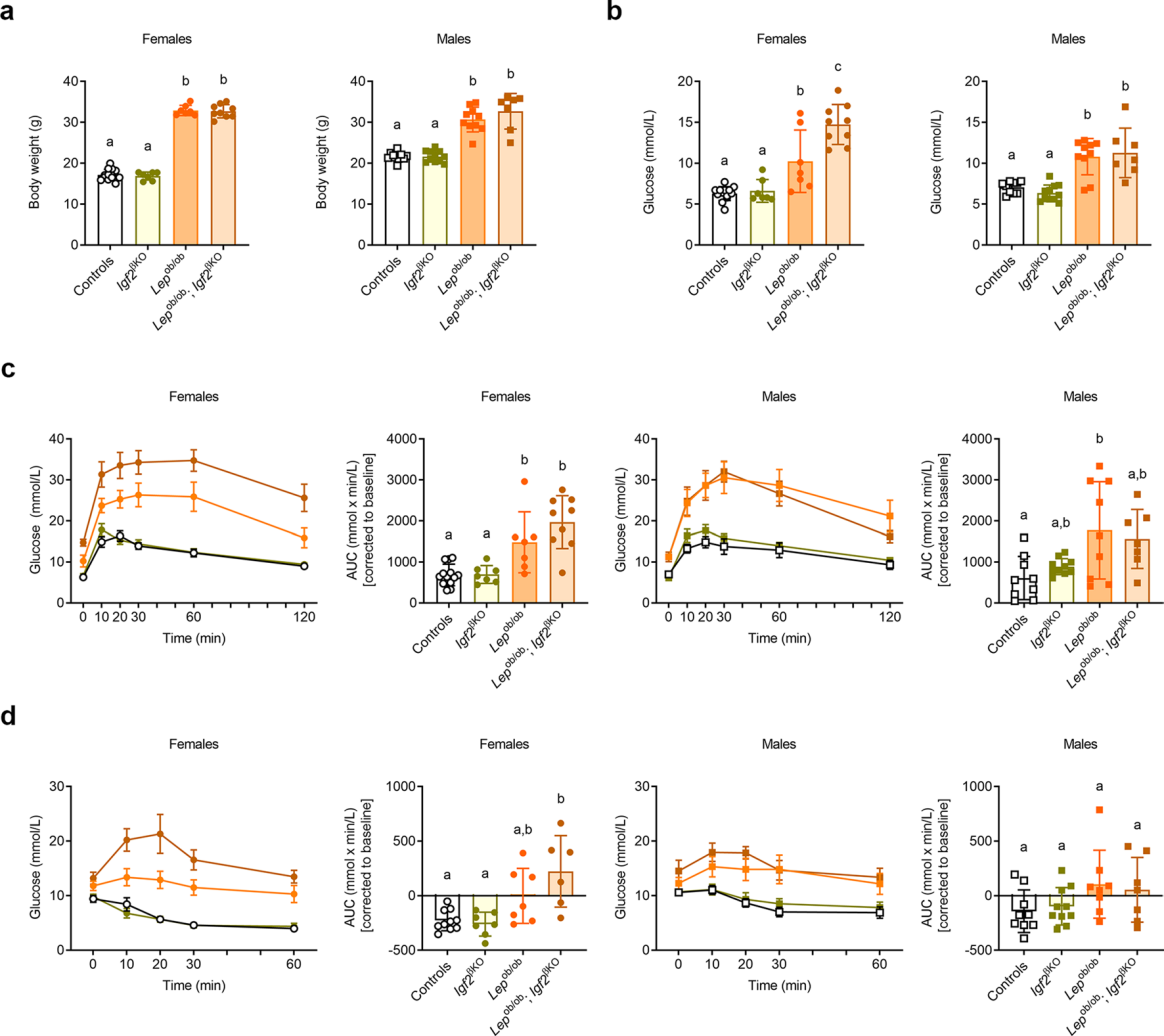
Impact of *Igf2*^βKO^ on body weight and glucose homeostasis in *Lep*^ob/ob^ in 8 week old male and female mice (**a**) Body weights. (**b**) Glucose levels in peripheral blood measured after overnight fasting. (**c**) ipGTTs performed after overnight fasting. First and third graphs show changes in blood glucose concentrations (y-axis), from basal pre-treatment values, with time (x-axis), after glucose administration. Second and fourth graphs show AUCs, calculated during ipGTT using the trapezoid rule, normalised to basal glucose levels. (**d**) ITTs performed after overnight fasting. First and third graphs show changes in blood glucose concentrations (y-axis), from basal pre-treatment values, with time (x-axis), after insulin administration. Second and fourth graphs show AUCs, calculated during ITT using the trapezoid rule, normalised to basal glucose levels. For all graphs, data is presented as individual values, with averages ± SD [panels (**a**) and (**b**) and second plus fourth graphs in panels (**c**) and (**d**)] or as averages ± SEM [first and third graphs in panels (**c**) and (**d**)]; n = 6–10 mice/group. Different letters indicate significant differences between groups (*P* < 0.05 by Tukey's post hoc test following one‐way ANOVA).

We next assessed the impact of *Igf2*^βKO^ mutation in a model of genetic obesity and marked insulin resistance due to congenital leptin deficiency (*Lep*^ob/ob^). *Lep*^ob/ob^ mice display severe obesity first recognisable at about four weeks of age^29^. We generated litters with four genotypes (controls, *Igf2*^βKO^, *Lep*^ob/ob^ and *Lep*^ob/ob^; *Igf2*^βKO^—Supplemental Fig. 5) and performed phenotypic analyses in young adult life (at the age of 8 weeks). For both sexes, mice in the *ob/ob* background were significantly obese, as expected, but there was no impact of *Igf2*^βKO^ mutation on overall body weight (Fig. 5a). Fasting glucose levels were also elevated in mice on the *ob/ob* background in both sexes (Fig. 5b). However, in females, but not in males, there was an additional significant increase in fasting glucose levels in *Lep*^ob/ob^; *Igf2*^βKO^ compared to *Lep*^ob/ob^ mice (Fig. 5b). For both sexes, ipGTTs showed significant glucose intolerance in mice on the *ob/ob* background (Fig. 5c). However, in both males and females, AUCs corrected to baseline were not statistically different between *Lep*^ob/ob^ and *Lep*^ob/ob^; *Igf2*^βKO^ mice (Fig. 5c). In females, we observed significant insulin resistance in mice on the *ob/ob* background, but with no further impact caused by the *Igf2*^βKO^ mutation (Fig. 5d). In males, all four genotypes had similar levels of insulin sensitivity, as assessed by AUC analyses after normalising to the baseline glucose levels (Fig. 5d). Together, these results demonstrate that lack of *Igf2* expression in pancreatic β-cells is detrimental in obese females with congenital leptin deficiency, leading to exacerbated hyperglycaemia in young adult life.

## Discussion

This study strongly suggests that autocrine IGF2 actions in pancreatic β-cells during early life program their capacity to adapt under circumstances that require increased insulin production in adult life, in females. We first show that under normal physiological conditions *Igf2*^βKO^ is compatible with normal pancreatic β-cell development, remodelling and function during early postnatal life, in both males and females. In the absence of a metabolic challenge, *Igf2*^βKO^ is also compatible with normal glucose homeostasis during adult life.

We then exposed *Igf2*^βKO^ and littermate control mice to several models of increased metabolic demand leading to insulin resistance and therefore need for increased insulin release. Firstly, we challenged *Igf2*^βKO^ via pregnancy, a physiological condition associated with reduced insulin sensitivity in the latter stages^20^. After six hours fasting, *Igf2*^βKO^ females became hyperglycaemic at E15, with normal levels of insulin and normal clearance of glucose from periphery during OGTT. These observations suggest that at this stage of pregnancy, *Igf2*^βKO^ females cannot reach the level of β-cell compensation required to maintain glucose homeostasis. We did not perform fasting glucose/insulin measurements or OGTTs at the end of gestation (E19). Therefore, we can’t rule out that, in the fasting state, *Igf2*^βKO^ females are hyperglycaemic at both time points or that they are glucose intolerant only at E19. However, we found that non-fasted *Igf2*^βKO^ females were normoglycaemic at both time points and exhibited hyperinsulinemia, in particular at E19, suggesting continued β-cell maladaptation. The effects observed in offspring provide evidence supporting the proposed maternal β-cell maladaptation. Accordingly, and although the conceptuses had normal development and growth up until at least E15, at E19 they associated placentomegaly and hyperinsulinemia. We did not identify the signal that triggers the observed changes in the conceptus. Given the known roles of placenta in mediating nutrient transfer between the mother and the fetus^30^, as well as the role played by several placental hormones in mediating maternal adaptations during pregnancy^31^, we propose that the observed changes in the mother and the fetus are interconnected. We speculate that the fetus is sensing intermittent episodes of maternal hyperglycaemia and adapting through increased production of insulin. In turn, fetal hyperinsulinemia elicit placentomegalia, with excessive secretion of placental hormones, perhaps leading to maternal hyperinsulinemia at the end of gestation. It would be interesting to assess in future studies whether the hyperinsulinemia observed in the fetuses of *Igf2*^βKO^ females at E19 is due to changes in fetal β-cell mass, or is caused by increased glucose-mediated insulin secretion, and has a long-lasting impact on their post-natal growth and metabolism. Similarly to our model, fetal hyperinsulinemia has been reported in liver-specific insulin receptor knockout mice (LIRKO), which results from sustained maternal hyperinsulinemia and transient increase in blood glucose concentrations during pregnancy^32^. Additionally, foetuses from mildly diabetic rat mothers (diabetes induced by streptozotocin administration prior to mating) show normal body weight, placentomegalia, higher pancreatic and plasma insulin concentrations at the end of gestation and enhanced insulin secretion by fetal pancreatic β-cells in response to glucose stimulation *in vitro*^33^. It would also be compelling to explore whether the metabolic changes observed during the first pregnancy in *Igf2*^βKO^ female mice become more pronounced in subsequent pregnancies, e.g. leading to diabetes. Women with gestational diabetes (GDM) have increased risk of recurrent GDM because of their underlying β-cell impairment^34^. Furthermore, previous GDM is an important predicting factor for subsequent diagnosis of diabetes in later life^35^.

The next model of increased metabolic demand used in this study was the exposure to a diet with high content in fat (HFD), known to induce obesity and insulin resistance^22^. We observed that *Igf2*^βKO^ females, but not the males, are more resistant to developing HFD-induced insulin resistance. However, the impact of *Igf2*^βKO^ mutation on insulin sensitivity in females fed HFD is relatively mild, with no impact on other variables measured. Additional tests such as hyperinsulinemic-euglycaemic glucose clamps, which allow a direct assessment of insulin resistance and glucose uptake by peripheral tissues^36^, would further refine the impact of *Igf2*^βKO^ on β-cell physiology in mice exposed to HFD. Our finding suggests the possibility of secreted signal(s) originating from pancreatic β-cells and affecting insulin sensitivity in the periphery. Our current knowledge of the β-cell secretome is limited^37,38^ but a very recent study uncovered the role of β-cell–derived exosomes that regulate peripheral insulin sensitivity in a paracrine manner via microRNA-26a (miR-26a)^39^. Future studies could explore the impact of autocrine IGF2 in regulating β-cell secretome.

An additional model of increased metabolic demand used in this study was that of congenital leptin deficiency^22^. In *Lep*^ob/ob^ mutants, *Igf2*^βKO^ led to augmented hyperglycaemia but only in females. It is interesting to observe that in two models of increased metabolic demand (congenital leptin deficiency and pregnancy), *Igf2*^βKO^ females exhibited hyperglycaemia, while in the third (HFD feeding) *Igf2*^βKO^ females were normoglycaemic and more insulin sensitive than their controls. We speculate that these outcome differences may relate to the diets used (chow diets are high in carbohydrates and low in lipids, while the HFD used in this study is high in lipids and low in carbohydrates) that trigger divergent insulin actions, as recently reviewed^40^. Based on the observations made in this study, we suggest that autocrine IGF2 may be more critical under circumstances with increased metabolic demand in females.

A number of previous studies proposed several molecular mechanisms by which autocrine IGF2 controls β-cell physiology. These range from protection against β-cell apoptosis (via a GLP-1-mediated increase in IGF1R expression)^41^, to promoting β-cell proliferation during pregnancy (dependent on estrogen, which reduces the expression of *miR-338-3p*, leading to increased IGF1R expression)^17^ and to controlling fasting insulin secretion (via IGF1R-AKT2-FAK signalling)^42^. However, these molecular mechanisms were based on observations made in vitro in insulin-secreting cell lines or in primary β-cells or pancreatic islets cultured ex vivo and require in vivo testing to be substantiated.

In a previous study, Modi et al*.* reported a more substantial impact of autocrine IGF2 on β-cell function using the same approach of a conditional deletion of *Igf2* in pancreatic β-cells^17^. Contrary to our observations, Modi et al*.* observed impaired β-cell expansion during pregnancy in *Igf2*^βKO^ females. Likewise, when fed HFD, they recorded significantly worsened glucose tolerance in *Igf2*^βKO^ females compared to controls. However, there are some significant differences between the experimental setup of the two studies, which may account for the phenotypic differences observed. First, we performed our experiments under a homogeneous C57BL/6 J genetic background. In contrast, Modi et al*.*^17^ used a mixed 129S6/C57BL/6 background. It is known that C57BL/6 and 129S6 strains exhibit notable differences in patterns of glucose homeostasis and insulin secretion under regular diet or when challenged with HFD^43^. Second, Modi et al*.*^17^ used *Ins1*-Cre to achieve deletion of *Igf2* in pancreatic β-cells, while we used *RIP*-Cre. *RIP*-Cre may have some weak ectopic activity in the brain^25^. However, as shown in the results section, we did not see any significant change of *Igf2* mRNA levels in the hypothalamus, and brain sections from offspring of *RIP*-Cre mice mated with *Rosa26YFP*-stop^fl/fl^ reporter mice showed only very few and sporadic YFP + cells. Additionally, in our experiments both control and mutant mice were all heterozygous for *RIP*-Cre, with maternal inheritance of the transgene. This design thus rules out a contribution of the Cre transgene on the observed mutant phenotypes. In most experiments reported by Modi et al*.*, only the mutants inherited an *Ins1*-Cre allele. Although hemizygosity for *Ins1* gene has no phenotypic impact on glucose homeostasis^44^, Cre recombinase expression has the potential to lead to changes in the pancreatic β-cells’ physiology (albeit the *Ins1*-Cre line has been carefully verified for lack of activity in the central nervous system and transmission of *Ins1*-Cre has been reported to not affect glucose homeostasis up until the age of 12 weeks^45^). Third, differences in HFD composition, age at feeding and length of exposure may be responsible for some of the diverging phenotypic outcomes. Indeed, Modi et al*.* performed the ipGTT challenge after a longer exposure to HFD diet (18 weeks compared to 12–14 weeks in the experiments reported in our study) and they used a diet with 45% energy derived from fat (60% in our study). The ages of various cohorts of mice used in the two studies were also distinct. Overall, these experimental differences call for caution when trying to draw direct comparisons between the two *Igf2*^βKO^ models. One of the common observations between our studies is that *Igf2*^βKO^ females seem more susceptible to develop altered glucose homeostasis than *Igf2*^βKO^ males when exposed to increased metabolic demand. Sex-related differences in β-cell function under stress conditions have been observed before in many clinical studies and in animal models of diabetes^46^. Estrogen acting on β-cells via ERα (estrogen receptor type α) promotes cell survival and insulin biosynthesis, and enhances GSIS through ERβ^47^. Interestingly, *Igf2* is a known estrogen-responsive gene, at least in some tissues such as the hippocampus^48^, and sex-related expression differences during development in organs such as the brain^49^ have been documented. Mody et al*.* proposed an interplay between estrogens and the IGF2 autocrine actions in controlling β-cell mass and function in female mice. Our results further suggest that *Igf2* may be one of the genes that mediate the protective actions of estrogen on pancreatic β-cells in females, under conditions of increased metabolic demand.

Given that IGF2 expression timing in mouse and human are different, is there any relevance for our findings regarding IGF2 actions in human β-cells? A significant number of genome-wide association studies (GWAS) have linked T2D with the human *INS*-*IGF2* locus^6,50^, as well as with the *IGF2BP2* locus^51^ that plays an important role in IGF2 mRNA translation^52^. A loss-of-function splice acceptor *IGF2* variant was found to protect against T2D^53^. None of the above studies can point directly to a defect in β-cell function. However, some studies provide a more direct link. Human pancreatic islets express a hybrid protein INS-IGF2 that consists of the pre-proinsulin signal peptide, the insulin B-chain, and eight amino acids of the C-peptide in addition to 138 amino acids encoded by the *IGF2* gene. *INS*-*IGF2* expression was lower in pancreatic islets of T2D donors compared to controls^54^. Notably, INS (38%), INS-IGF2 (10%) and IGF2 (2%) were the top three most abundant transcripts expressed in human β-cells isolated from donors without diabetes^55^. Finally, some forms of insulinomas, endocrine pancreatic tumours that lead to severe forms of hyperinsulinemic hypoglycaemia, associated hypermethylation at the *IGF2* differentially methylated region 2 (*IGF2*-DMR2), with *IGF2* loss-of-imprinting and overexpression^56^.

Our study has a number of limitations. First, we cannot fully exclude that the *RIP*-Cre line used in our study has an impact on β-cell physiology that could influence the differences observed when comparing controls to *Igf2*^βKO^ mutants. Other β-cell Cre drivers have been previously shown to induce impaired islet function due to the expression of a human growth hormone (hGH) minigene, which was frequently used to enhance transgene activity^57^. The *RIP*-Cre line used in this study does not contain the hGH minigene^58^. However, a more subtle impact on β-cell function may still exist. Second, we cannot separate, in our study, autocrine IGF2 actions in early life, when *Igf2* mRNA levels are much higher, from those in adult life, when *Igf2* mRNA levels are very low, but still detectable. A direct proof that the observed phenotypes are due to programming effects of autocrine IGF2 in early life, as suggested, would require the use of inducible β-cell-specific Cre lines that enable temporal control of Cre recombination^59^. Third, we cannot exclude a contribution of *miR-483*, a microRNA embedded within intron 4 of *Igf2* and deleted in our model, to the phenotypes observed in *Igf2*^βKO^ mutants. Previous in vitro data obtained in MIN6 insulin-secreting cells, has shown that *miR-483* promotes insulin transcription and secretion by targeting SOCS3, a member of suppressor of cytokine signalling family^60^. Fourth, we did not explore the impact of *Igf2*^βKO^ in the context of ageing, which is one of the well-known risk factors for type 2 diabetes development^61^. Aged mice are considered to be those older than 18 months, which has been suggested to be equivalent to 56 + in human years^62^. In the cohorts of mice fed normal chow or HFD, we did not extend the follow-up beyond 10 months. We cannot exclude that *Igf2*^βKO^ would develop diabetes when significantly older. Fifth, for the cohort of mice fed HFD, the phenotypic studies were conducted after 12–14 weeks of exposure, similar to timelines reported in other studies using conditional deletions in pancreatic β-cells^63–65^. However, we cannot exclude that more prolonged HFD feeding is required for *Igf2*^βKO^ mice to reach their maximum capacity for β-cell mass expansion, leading to more severe glucose intolerance compared to controls. Sixth, combinations of stress conditions, such as pregnancy associated with HFD feeding, or ageing and HFD feeding may be required to uncover β-cell functional defects induced by the *Igf2*^βKO^ mutation. Lastly, the autocrine IGF2 actions may be masked by the paracrine actions of IGF2 produced by neighbouring cells. In our previous study we observed that mice lacking mesenchyme-derived IGF2 have reduced β-cell mass and develop glucose intolerance during pregnancy^18^. We also observed significant expression of *Igf2* mRNA in the endothelial cells. Other endocrine cells within the islets of Langerhans may also affect β-cell plasticity via paracrine IGF2 actions. Combinations of conditional deletions in multiple cell types, aimed at reducing the paracrine IGF2 actions may uncover additional roles of autocrine IGF2 in pancreatic β-cells.

In summary, we report in this study that autocrine actions of IGF2, although not required for development of pancreatic β-cells, have a long-term impact on β-cell plasticity that becomes apparent in female mice under conditions associated with increased demand for insulin. Our results also highlight that even subtle defects in maternal pancreatic β-cell function can affect the normal development and physiology of the descendants, with potential implication for metabolic health in later life.

## Methods

### Ethics statement

This study was carried out in compliance with the ARRIVE guidelines^66^. The research has been regulated under the Animals (Scientific Procedures) Act 1986 Amendment Regulations 2012 following ethical review and approval by the University of Cambridge Animal Welfare and Ethical Review Body (AWERB) and by the Ethical and Veterinary committees of Rey Juan Carlos University in Spain. All mouse experiments were approved and performed under PPL No. 80/2483 (study 2483/02/12), PPL No. 80/2484 (study 2484/38/12) and PPL No. 70/7594 (study 7594/4/15).

### Mouse strains and husbandry

*Igf2*^fl/+^ mice were generated in our laboratory, as described^18^. C57BL/6J mice were purchased from Charles River (Kent, UK). *Rosa26YFP*-stop^fl/fl^ reporter mice^24^ were kindly provided by Dr. Martin Turner (The Babraham Institute, Cambridge). *RIP*-Cre mice that carry a Cre transgene under the control of the rat *Ins2* (insulin 2) promoter (RIP), which directs expression to insulin-positive β-cells from approximately E8.5–9 onwards^23^, were obtained from Central Biomedical Services (CBS Transgenic Services, University of Cambridge). *Lep*^ob/+^ mice that have a spontaneous mutation in the *Lep* gene encoding leptin^67^ were available at the Universidad Rey Juan Carlos mouse facility. All lines were maintained onto an inbred C57BL/6J genetic background for > 10 generations prior to the experiments performed in this study.

Mice were fed a standard chow diet with 9% of kcal from fat (SDS, Essex, UK), or a high fat diet (HFD) containing 60% kcal from fat (D12492, Research diets Inc., New Brunswick, USA) and housed with a 12-h light/dark cycle in a temperature-controlled room (22 °C). Food and water were available ad libitum, except for periods of fasting when food was withdrawn. For timed matings, the day of detection of a vaginal plug was noted as embryonic day 1 (E1) and the day of birth was noted as post-natal day 0 (P0). Mice were weaned at 3 weeks of age and ear notches were used for visual identification and genotyping, which was performed using standard PCR or qPCR (quantitative PCR—in order to discriminate between *RIP*^Cre/+^ and *RIP*^Cre/Cre^) with primers listed in Supplementary Table 1. Genotyping for the mouse obese (*ob*) mutation at the *Lep* locus was performed by PCR and restriction fragment length polymorphism (RFLP) analysis, as described^68^.

### Fluorescence-activated cell sorting (FACS)

P2 pups were sacrificed by decapitation. Then, pancreases were dissected under a dissection microscope and dissociated into single cells with trypsin–EDTA (Sigma Aldrich), at 37 °C, for 20 min. After washing with ice-cold PBS, cells were passed through 70 μm strainers and single-cell suspensions were sorted into YFP^+^ and YFP^-^ fractions using an Aria-Fusion cell sorter (BD Bioscience). Dead cells were excluded based on forward and side scatter profiles and the uptake of 7AAD (7-Aminoactinomycin D dead cell stain, Life Technologies). Sorted YFP^+^ cells were pelleted by centrifugation and flash frozen using liquid nitrogen (N2), and then stored at -80 °C until use.

### qRT-PCR analysis

Total RNA was extracted from FACS-isolated β-cells and other organs using RNeasy Plus Kits (Qiagen—74134 and 74034). RNA concentration was measured by NanoDrop (Thermo Scientific) and quality was assessed in agarose gels. RNA extracted from FACS-isolated β-cells was quantified and assessed for quality using the RNA 6000 Pico Kit (Agilent—5067-1513) and an Agilent 2100 Bioanalyzer. Reverse transcription was performed using the RevertAid RT Reverse Transcription Kit (ThermoFisher—K1622). In the case of total RNA extracted from FACS-isolated β-cells, cDNA was produced using the QuantiTect Whole Transcriptome Kit (Qiagen) following manufacturer’s instructions. qRT-PCR was performed with the SYBR Green JumpStart Taq Ready Mix (Sigma—S4438) and custom-made primers (Supplementary Table 2) using an ABI Prism 7900 system (Applied Biosystems). Gene expression normalisation was performed against three housekeeping genes: *Ppia* (peptidylpropyl isomerase A or cyclophilin-A), *Gapdh* (glyceraldehyde 3-phosphate dehydrogenase) and *Sdha* (succinate dehydrogenase complex flavoprotein subunit A). Relative levels of expression were calculated using the 2^−ΔΔCt^ method^69^.

### Immunostainings, cell counting and β-cell mass analyses

Pancreases and brains were dissected (using a stereoscope for the early post-natal analyses), fixed in 4% paraformaldehyde in PBS overnight, dehydrated and then embedded in paraffin. Paraffin blocks were cut at 5 μm thickness, sections were then deparaffinised, rehydrated, stained and mounted with coverslips. Insulin and YFP stains and β-cell mass measurements using pancreas stereology were performed as previously described^18^. Measurements of β-cell proliferation were preceded by intraperitoneal injections with 50 µg of 5-ethynyl-2′-deoxyuridine (EdU)/g body weight, 6 h prior to tissue collection. EdU staining was done using the Click-iT EdU Alexa Fluor 488 Imaging Kit (Invitrogen—C10337), according to manufacturer’s instructions. TUNEL staining was performed using the In Situ Cell Death Detection Kit, TMR red (Sigma—012156792910), according to manufacturer’s protocol. For immunofluorescence stains, Hoechst33342 (Sigma—B2261) or DAPI (Sigma—D9542) were used to label the nuclei. Immunofluorescence image acquisition was performed as Z-stacks using a LSM510 Meta confocal laser scanning microscope (Carl Zeiss, Jena, Germany) and the ZEN 2009 software. Counting proliferating β-cells (EdU^+^/INS^+^) and apoptotic β-cells (TUNEL^+^/INS^+^) was performed using Volocity 6.3 (Improvision).

### Body composition

Body composition analysis was performed by time-domain nuclear magnetic resonance spectroscopy (TD-NMR) that measures total body fat mass and lean mass^70^. For this purpose, live and conscious mice were placed inside the Minispec Live Mice Analyser (Bruker Minispec Live Mice Analyser LF50).

### Glucose and insulin tolerance tests

Oral glucose tolerance tests (OGTTs), intra-peritoneal glucose tolerance tests (ipGTTs) and insulin tolerance tests (ITTs) were performed on conscious mice after 6 h fasting (8am to 2 pm—for OGTTs performed on pregnant females, for OGTTs and ITTs on mice fed HFD, and for ITTs for the cross with *Lep*^ob/+^ mice) or 16 h fasting (5 pm to 9am following day—for ipGTTs performed on adult mice fed chow diet and for the cross with *Lep*^ob/+^ mice). Throughout these experiments, mice were kept in heated cages (32 °C) to facilitate blood collection from the tail vein. Blood samples, taken from the tail vein immediately before the start of each experiment, were used to measure glucose and/or insulin levels in the fasting state. For OGTT, glucose was administered by oral gavage at a dose of 2 mg/g body weight (adjusted per individual animal for the pregnancy experiment or fixed volume calculated for an averaged body weight for the HFD experiment). For ipGTTs, glucose was administered by i.p. injection at a dose of 1 mg/g body weight. For ITTs performed on mice fed HFD, insulin was administered by i.p. injection at a dose of 0.75mUI/g body weight for females and 1mUI/g body weight for males (fixed volume calculated for an averaged body weight). For ITTs performed for the cross with *Lep*^ob/+^ mice, same dose of 0.75mUI/g body weight was used for both sexes. Throughout the experiments, glucose measurements were performed using a glucose meter and test strips (AlphaTRAK). The areas under the curve (AUCs) following OGTTs, ipGTTs or ITTs were calculated by the trapezoidal rule.

### Plasma insulin and total pancreas insulin measurements

Blood samples for plasma insulin measurements were collected in heparinised capillary tubes during OGTT experiments at 0, 15, 30, 45 and 60 min. Tubes were kept on ice and spun at 4,000 RPM (rotations per minute) for 5 min. Plasma samples were flash frozen in liquid N2 and stored at -80 °C until analysis. For total pancreas insulin measurements, whole pancreases were flash frozen in liquid N2, then pulverised and re-suspended in cold acid–ethanol and stored at 4 °C for 48 h, with sonication every 24 h during the storage. Insulin levels in plasma and acid–ethanol supernatants were measured using ELISA kits (Meso Scale Discovery Mouse/Rat Insulin Assay Kit) at CBAL (Core Biochemical Assay Laboratory, Addenbrooke’s hospital). Total pancreas insulin content (ng) was normalised to the total pancreas wet weight (mg), measured at collection.

### Blood biochemistry

Serum glucose, triglycerides, free (non-esterified) fatty acids, and total cholesterol concentrations were measured using enzymatic assay kits. Briefly, glucose was measured based on an adaptation of the hexokinase-glucose-6-phosphate dehydrogenase method using a kit from Siemens Healthcare (product code DF30). Triglycerides were measured using an enzymatic assay kit from Siemens Healthcare (product code DF69A) that combines activities of lipoprotein lipase, glycerol kinase and glycerol-3-phosphate oxidase. Total cholesterol was measured using an enzymatic assay kit from Siemens Healthcare (product code DF27) that combines activities of cholesterol esterase and cholesterol oxidase. The assays for glucose, triglycerides and total cholesterol were automated on the Siemens Dimension EXL analyser. Free (non-esterified) fatty acids were measured using Roche’s Free Fatty Acid Kit (half-micro test) (Sigma Aldrich product code 11383175001) that is based on the enzymatic conversion of free fatty acids to acyl CoA by acyl-Co A synthetase. Leptin, adiponectin and resistin measurements were performed using enzyme-linked immunosorbent assay kits manufactured by MesoScale Discovery (MSD) Rockville, MD, USA, on a MSD s600 instrument, according to manufacturer instructions (K152BYC kit for leptin, K152BXC kit for adiponectin and K152FNC kit for resistin). All blood biochemistry measurements were performed at CBAL, Addenbrooke’s hospital.

### Statistical analyses

Statistical analyses were performed using GraphPad Prism 8 software. For two groups, statistical analyses were performed using Mann–Whitney tests or un-paired Student’s *t*-tests with Welch’s correction (depending on the outcome of Shapiro–Wilk tests for normal distribution). Where more than two groups were analysed, we used one-way ANOVA, followed by Tukey’s multiple comparisons tests or two-way ANOVA followed by Sidak’s corrections for multiple testing, as appropriate. For growth kinetics analyses, we used mixed-effects model (REML) tests. For all tests, *P* values < 0.05 were considered significant. Detailed results of statistical analyses are shown in Supplementary Table 3.

## Supplementary Information

Supplementary Information
