# Supplementary material for: Autocrine IGF2 programmes β-cell plasticity under conditions of increased metabolic demand
Source: Sci Rep. 2021 Apr 8;11:7717. doi: 10.1038/s41598-021-87292-x (PMC8032793; doi:10.1038/s41598-021-87292-x)
Supplement: Supplementary file 1 — Supplementary Information [file 41598_2021_87292_MOESM1_ESM.docx]

**SUPPLEMENTARY INFORMATION**

**Title: Autocrine IGF2 programmes β-cell plasticity under conditions of increased metabolic demand**

Ionel Sandovici^1,2,3,^*^,✉^, Constanze M. Hammerle^1,2,§,^*^,✉^, Sam Virtue^1^, Yurena Vivas-Garcia^4,§§^, Adriana Izquierdo-Lahuerta^4^, Susan E. Ozanne^1^, Antonio Vidal-Puig^1,5,6^, Gema Medina-Gómez^4^ & Miguel Constância^1,2,3,✉^

^1^ University of Cambridge Metabolic Research Laboratories and MRC Metabolic Diseases Unit, Institute of Metabolic Science, Addenbrookes Hospital, Cambridge CB2 0QQ

^2^ Department of Obstetrics and Gynaecology and National Institute for Health Research Cambridge Biomedical Research Centre, Cambridge CB2 0SW, United Kingdom

^3^ Centre for Trophoblast Research, Department of Physiology, Development and Neuroscience, University of Cambridge, Cambridge CB2 3EG, United Kingdom

^4^ Área de Bioquímica y Biología Molecular, Departamento de Ciencias Básicas de la Salud, Universidad Rey Juan Carlos, 28922-Alcorcón, Madrid, Spain

^5^ Welcome Trust Sanger Institute, Hinxton, CB10 1SA, United Kingdom

^6^ Cambridge University Nanjing Centre of Technology and Innovation, Jiangbei Area, Nanjing, PR China

^§^ Present address: Novo Nordisk A/S, DK-2880 Bagsværd, Denmark

^§§^ Present address: Ludwig Institute for Cancer Research, Nuffield Department of Clinical Medicine, University of Oxford, Headington, Oxford OX3 7DQ, United Kingdom

* These authors contributed equally to this work

^✉^ e-mails: [is299@cam.ac.uk](mailto:is299@cam.ac.uk); [czmh@novonordisk.com](mailto:czmh@novonordisk.com); [jmasmc2@cam.ac.uk](mailto:jmasmc2@cam.ac.uk)


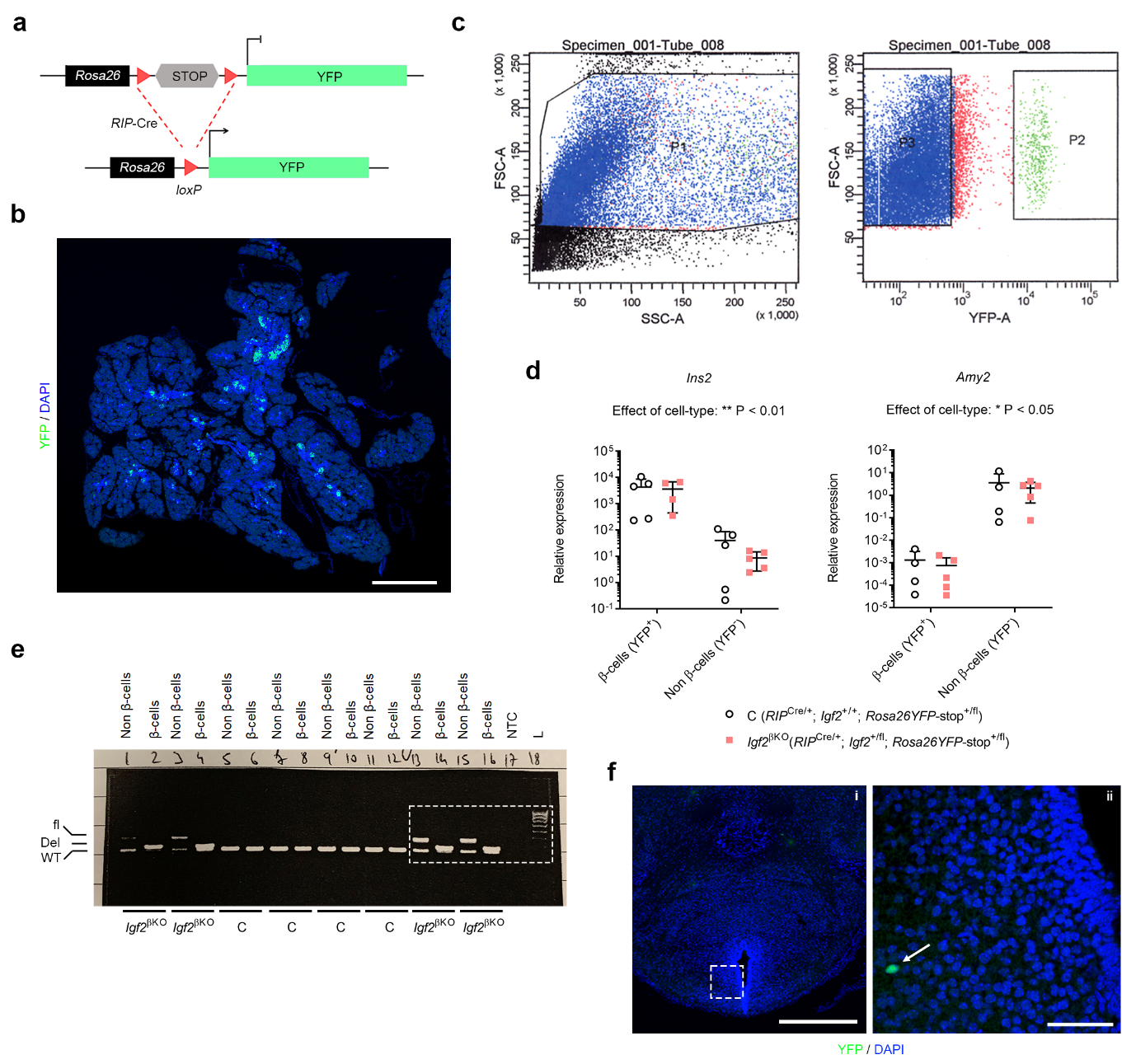


**Supplemental Figure S1.** Assessment of *Igf2* deletion specificity in pancreatic β-cells by *RIP*-Cre using *Rosa26YFP*-stop^fl/fl^ reporter mouse. (**a**) Schematic representation of *Rosa26YFP*-stop floxed allele before and after removal of the stop cassette by *RIP*-Cre, leading to YFP production in pancreatic β-cells. (**b**) Representative staining for YFP of a pancreas section collected from *RIP*^Cre/+^; *Rosa26YFP*-stop^+/fl^ post-natal day 2 (P2) pups. DAPI staining (blue) labels the nuclei. Scale bar is 500 µm. (**c**) Representative FACS gating used for isolation of YFP+ pancreatic β-cells (fraction P2) at postnatal day 2. (**d**) Expression levels of *Ins2* (marker of pancreatic β-cells) and *Amy2* (marker of acinar cells) measured by qRT-PCR in FACS sorted cells demonstrate significant enrichment of pancreatic β-cells in the YFP+ fraction (note the logarithmic scale for the Y-axis). Levels are normalised against *Ppia*, *Gapdh* and *Sdha*, used as internal controls (n = 4 – 5 samples/group). Data is shown as individual values, with averages ± SD. *P* values shown above the graphs were calculated by two-way ANOVA tests. (**e**) Original PCR gel picture used to generate Fig. 1a (the portion presented in Fig. 1a is indicated with a dotted white line). WT – wild-type allele (observed in both C – controls and *Igf2*^βKO^ mutants); Del – deletion allele (observed only in the β-cell fraction of *Igf2*^βKO^ mutants); fl – floxed allele (observed only in Non β-cell fraction of *Igf2*^βKO^ mutants); NTC – no template control; L – 100 bp DNA ladder. (**f**) Representative staining for YFP of a coronal section through hypothalamus collected at P2 from *RIP*^Cre/+^ ; *Rosa26YFP*-stop^+/fl^ pups (i). The dotted-line square corresponds to the image shown at higher magnification in panel (ii). DAPI staining (blue) labels the nuclei. Scale bars are: 500 µm for panel (i) and 50 µm for panel (ii). Arrow points towards an isolated YFP+ hypothalamic neuron.


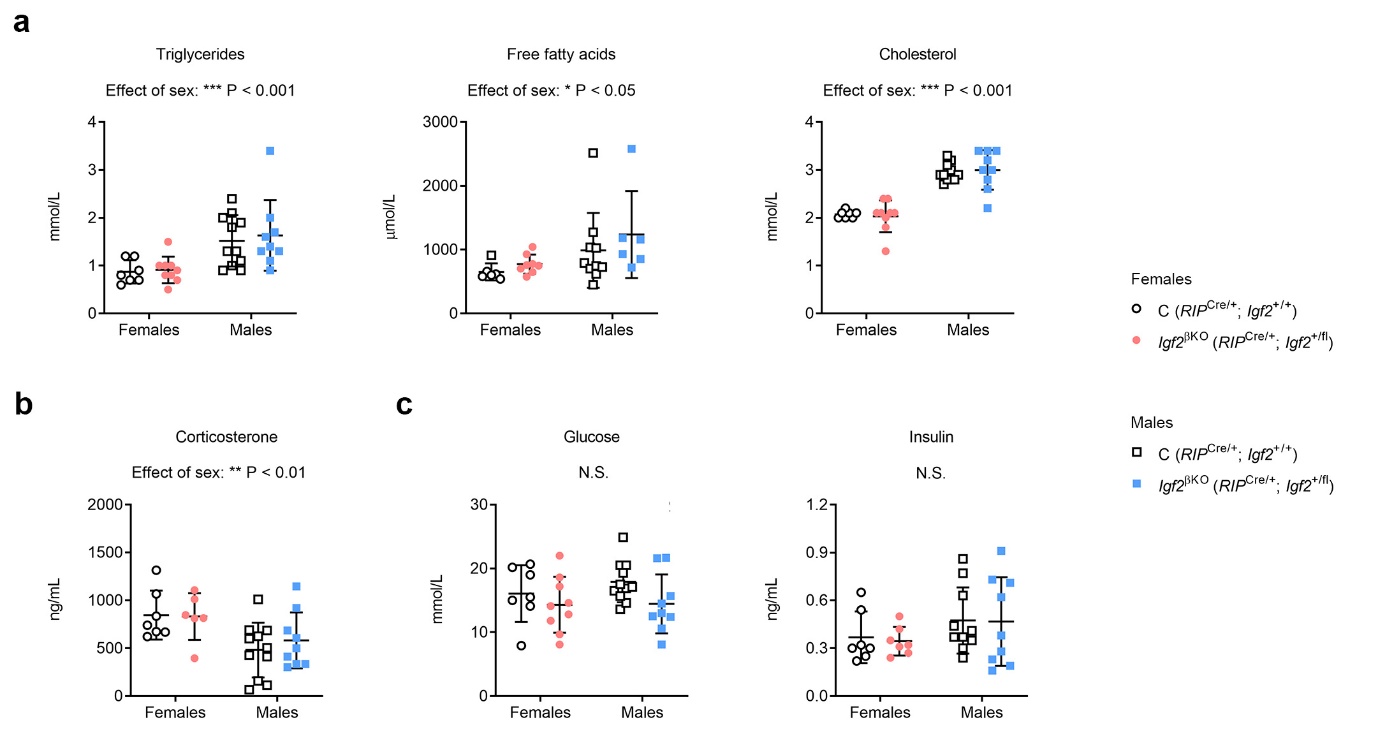


**Supplemental Figure S2.** Blood biochemistry analyses in 48 week old C and *Igf2*^βKO^ mice after an overnight fast. (**a**) Lipid profile, (**b**) Levels of the stress hormone corticosterone and (**c**) Markers of glucose homeostasis measured in serum collected after overnight fasting. For all panels, data is shown as individual values with averages ± SD (n = 6 – 11 samples/group). *P* values above the graphs were calculated by two-way ANOVA tests; N.S. – non-significant.


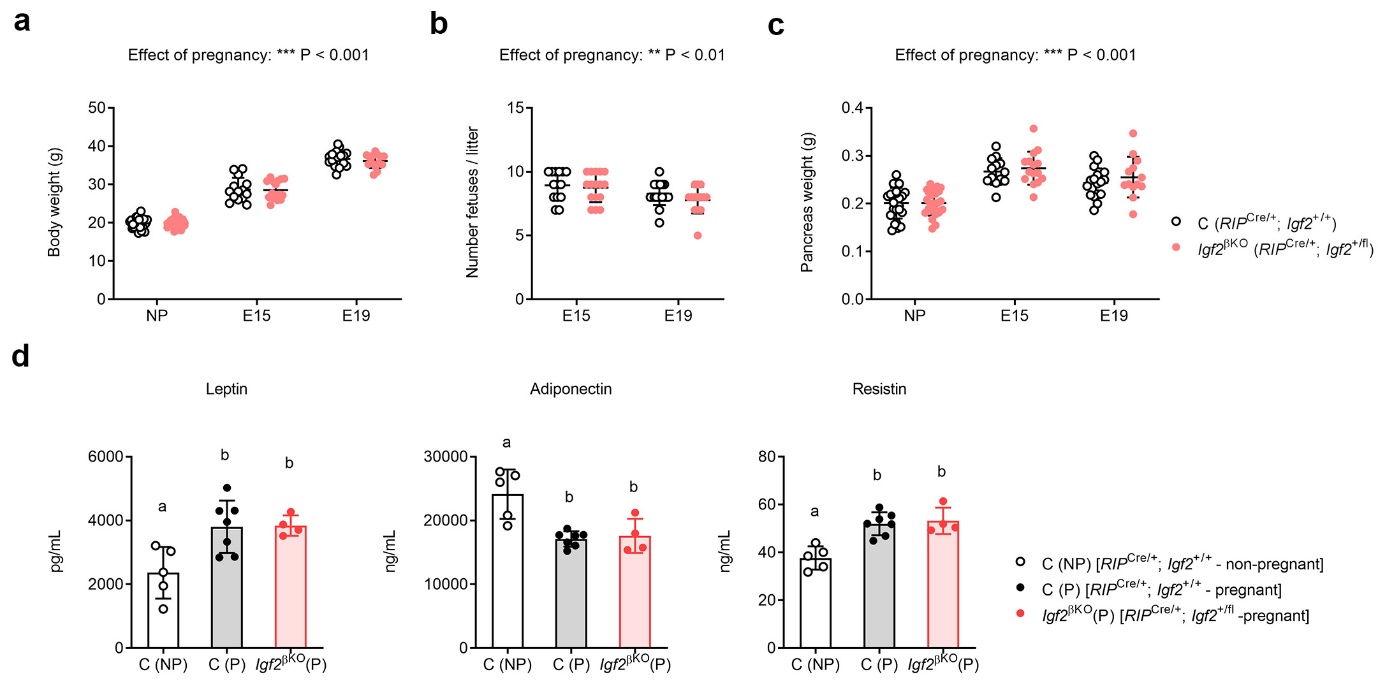


**Supplemental Figure S3.** Weights and molecular markers of pregnancy-related insulin resistance measured in *Igf2*^βKO^ females and control littermates. (**a**) Pregnancy-associated body weight gain at embryonic day 15 (E15) and E19 of gestation, compared to age-matched non-pregnant females (NP) (n = 14 – 25 females/group). (**b**) Litter sizes at E15 and E19 (n = 14 – 19 litters/group). (**c**) Pregnancy-associated pancreas weight gain at E15 and E19 compared to age-matched NP females (n = 13 – 25 samples/group). (**d**) Markers of pregnancy-associated insulin resistance measured in the non-fasted state at E19 of gestation (P) compared to age-matched non-pregnant controls (NP) (n = 4 – 7 samples/group). Different letters indicate significant differences between groups. For all panels data is shown as individual values, with averages ± SD. *P* values shown above the graphs were calculated using two-way ANOVA tests [panels (**a**), (**b**) and (**c**)] or by one-way ANOVA with Tukey’s correction for multiple testing [panel (**d**)].


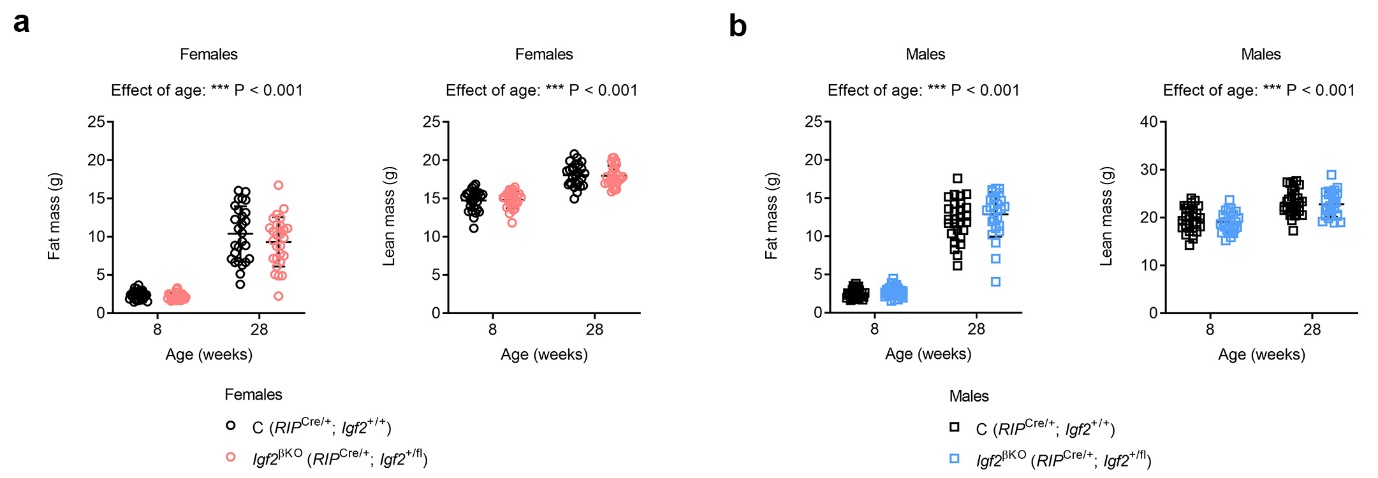


**Supplemental Figure S4.** Age-related changes in body composition. TD-NMR analyses in females (**a**) and males (**b**) fed chow diet. For all panels, data is shown as individual values, with averages ± SD. *P* values shown above the graphs were calculated by two-way ANOVA tests (n = 27 – 28 mice/group).


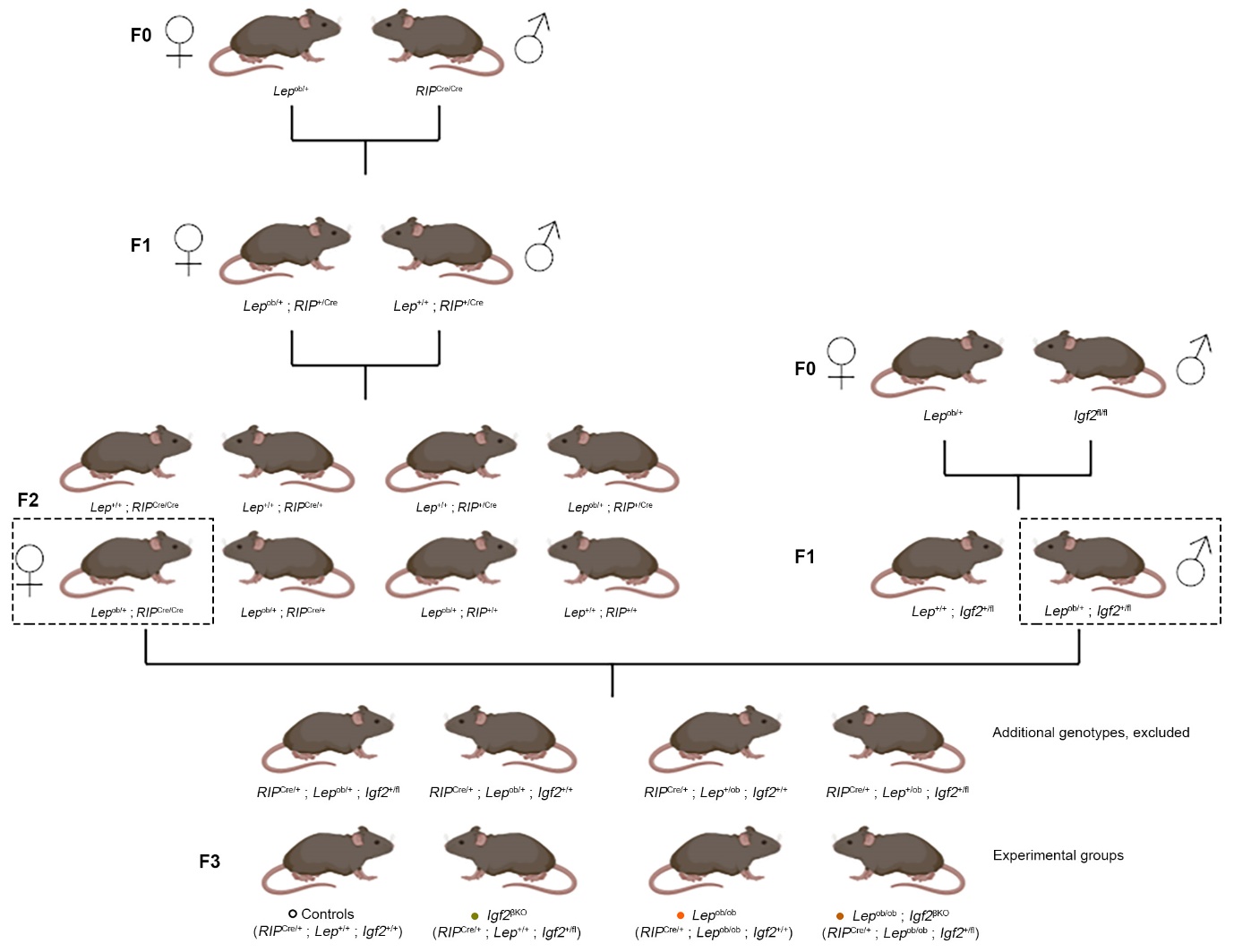


**Supplemental Figure S5.** Breeding strategy employed for generating *Lep*^ob/ob^; *Igf2*^βKO^ double mutants and associated experimental groups. Left side: In F0, *Lep*^ob/+^ females were mated with *RIP*^Cre/Cre^ males to generate *Lep*^ob/+^ ; *RIP*^+/Cre^ offspring. In F1, *Lep*^ob/+^ ; *RIP*^+/Cre^ females and *Lep*^+/+^ ; *RIP*^+/Cre^ males were inter-crossed to generate offspring with eight possible genotypes, including *Lep*^ob/+^ ; *RIP*^Cre/Cre^ females (dotted line box). Right side: In F0, *Lep*^ob/+^ females were mated with *Igf2*^fl/fl^ males to generate *Lep*^ob/+^ ; *Igf2*^+/fl^ offspring, including males (dotted line box). *Lep*^ob/+^ ; *RIP*^Cre/Cre^ females (dotted line box on the left) were mated with *Lep*^ob/+^ ; *Igf2*^+/fl^ males (dotted line box on the right) to generate the four experimental genotypes (shown with coloured dots), alongside four additional genotypes (all heterozygous for the *Lep*^ob/+^ mutation – excluded from phenotypic analyses). Note that all four experimental groups, including controls, are heterozygous *RIP*^Cre/+^ (image generated using BioRender [https://app.biorender.com]).

**Supplementary Table 1.** **Primers used for genotyping by PCR or qPCR.**

| Strain | Primer | Sequence (5’ to 3’) | Primer | Sequence (5’ to 3’) | Amplicon (bp) |
| --- | --- | --- | --- | --- | --- |
| *Igf2*^+/fl^ | F | TTACAGTTCAAAGCCACCACG | RW  RD | GCCAAAGAGATGAGAAGCACC  GCCAAACACAGTAAAAAGAAATGC | WT: 324  fl: 449  Del: 384 |
| *RIP*-Cre  (standard PCR) | F-WT  F-Cre | ATGTCTCCAATCCTTGAACACTG  CGAGTGATGAGGTTCGCAAG | R-WT  R-Cre | GCAGTGGGAGAAATCAGAACC  TGAGTGAACGAACCTGGTCG | WT: 254  Cre: 390 |
| *RIP*-Cre  (qPCR) | F-Cre  Csa-F | CGAAAAAGAAAGAACAATCAAGGG  TGGTTGGCATTTTATCCCTAGAAC | R-Cre  Csa-R | AAACAGCAAAGTCCAGGGGTC  GCAACATGGCAACTGGAAACA | Cre: 164  Csa: 134  (internal control) |
| *Rosa26YFP-*stop^+/fl^ | F | TGTTATCAGTAAGGGAGCT | R-WT  R-fl | CACACCAGGTTAGCCTTTA  AAGACCGCGAAGAGTTTGT | WT: 239  fl: 301 |

+ or WT – wild-type allele; fl – floxed allele; Del – deletion.

**Supplementary Table 2.** **Primers/assays used for qRT-PCR.**

| Gene | Forward primer (5’ to 3’) | Reverse primer (5’ to 3’) | Amplicon (bp) |
| --- | --- | --- | --- |
| *Igf2* | AGTCCGAGAGGGACGTGTCTA | CGGACTGTCTCCAGGTGTCAT | 102 |
| *Ppia* | AAGGGTTCCTCCTTTCACAGAA | GATGCCAGGACCTGTATGCTT | 146 |
| *Gapdh* | ACAACTCACTCAAGATTGTCAGCA | ATGGCATGGACTGTGGTCAT | 121 |
| *Sdha* | TTCCGTGTGGGGAGTGTATTG | ATTCTGCAGCTCCAGGGTCTC | 135 |
| *Ins2* | GCGTGGCTTCTTCTACACACC | CAGTGCCAAGGTCTGAAGGTC | 112 |
| *Amy2* | GCAAGTGGAATGGCGAGAAG | TCGCTGATTATCATGGTTGTCC | 110 |

**Supplementary Table 3. Detailed results of statistical analyses.**

| Figure | Statistical test | P values |
| --- | --- | --- |
| 1b | Multiple *t* tests with Holm-Sidak correction for multiple testing | Liver: P = 0.5199  Kidney: P = 0.9868  Spleen: P = 0.9767  Hypothalamus: P = 0.9767  β-cells: P < 0.0001 |
| 1d | Two-way ANOVA  Sidak's multiple comparisons tests | P age < 0.0001  P genotype = 0.0199  P interaction = 0.7102  P5: P = 0.4763  P10: P = 0.1381  P14: P = 0.7752 |
| 1e | Two-way ANOVA  Sidak's multiple comparisons tests | P age < 0.0001  P genotype = 0.6549  P interaction = 0.2340  P5: P > 0.9999  P10: P = 0.2264  P14: P = 0.8808 |
| 1f | Two-way ANOVA  Sidak's multiple comparisons tests | P age < 0.0001  P genotype = 0.2686  P interaction = 0.0545  P5: P = 0.7762  P14: P = 0.0729 |
| 1g | Mann-Whitney test | P = 0.6831 |
| 1h | Two-way ANOVA  Sidak's multiple comparisons tests | P age < 0.0001  P genotype = 0.3568  P interaction = 0.8777  P5: P = 0.9832  P10: P = 0.9848  P14: P = 0.6100 |
| 2a | Mixed-effects model tests | Females:  P age < 0.0001  P genotype = 0.3553  P interaction = 0.1897  Males:  P age < 0.0001  P genotype = 0.2511  P interaction = 0.4539 |
| 2b | Two-way ANOVA  Sidak's multiple comparisons tests | P age = 0.4082  P genotype = 0.0727  P interaction = 0.0994  4 weeks: P = 0.0637  17 weeks: P = 0.8509  39 weeks: P = 0.9609 |
| 2c | Two-way ANOVA  Sidak's multiple comparisons tests | P age = 0.0035  P genotype = 0.7603  P interaction = 0.1283  4 weeks: P = 0.6916  17 weeks: P = 0.3463  39 weeks: P = 0.6917 |
| 3a | Two-way ANOVA  Sidak's multiple comparisons tests  Two-way ANOVA  Sidak's multiple comparisons tests | Glucose:  P pregnancy = 0.0057  P genotype = 0.4379  P interaction = 0.9467  NP: P = 0.9948  E15: P = 0.9181  E19: P = 0.9310  Insulin:  P pregnancy < 0.0001  P genotype = 0.0045  P interaction = 0.0601  NP: P = 0.9968  E15: P = 0.1077  E19: P = 0.0041 |
| 3b | Unpaired *t* test with Welch’s correction | P = 0.2783 |
| 3c | Mann-Whitney tests | Fasting glucose: P = 0.0081  Fasting insulin: P = 0.7232 |
| 3d | Unpaired *t* test with Welch's correction | P = 0.2483 |
| 3e | Two-way ANOVA  Sidak's multiple comparisons tests  Two-way ANOVA  Sidak's multiple comparisons tests | Fetus:  P stage < 0.0001  P genotype = 0.1656  P interaction = 0.8298  E15: P = 0.6442  E19: P = 0.4537  Placenta:  P stage < 0.0001  P genotype = 0.0002  P interaction = 0.3177  E15: P = 0.0943  E19: P = 0.0017 |
| 3f | Two-way ANOVA  Sidak's multiple comparisons tests  Mann-Whitney test | Glucose:  P stage < 0.0001  P genotype = 0.1265  P interaction = 0.5305  E15: P = 0.2283  E19: P = 0.7788  Insulin:  P < 0.0001 |
| 4a | Mixed-effects model tests | Females:  Age: P < 0.0001  Diet: P < 0.0001  Genotype: P = 0.2246  Age x Diet: P < 0.0001  Age x Genotype: P = 0.2270  Diet x Genotype: P = 0.2784  Age x Diet x Genotype: P = 0.6995  Males:  Age: P < 0.0001  Diet: P = 0.0032  Genotype: P = 0.6343  Age x Diet: P < 0.0001  Age x Genotype: P = 0.9612  Diet x Genotype: P = 0.7094  Age x Diet x Genotype: P = 0.9896 |
| 4b | Two-way ANOVA  Sidak's multiple comparisons tests  Two-way ANOVA  Sidak's multiple comparisons tests | Females:  P diet < 0.0001  P genotype = 0.2407  P interaction = 0.4072  Chow: P = 0.9604  HFD: P = 0.3050  Males:  P diet < 0.0001  P genotype = 0.4682  P interaction = 0.1294  Chow: P = 0.8166  HFD: P = 0.2107 |
| 4c | Two-way ANOVA  Sidak's multiple comparisons tests  Two-way ANOVA  Sidak's multiple comparisons tests | Females:  P diet = 0.0075  P genotype = 0.3241  P interaction = 0.6473  Chow: P = 0.5086  HFD: P = 0.9168  Males:  P diet = 0.6222  P genotype = 0.2189  P interaction = 0.1423  Chow: P = 0.9814  HFD: P = 0.1105 |
| 4d | Two-way ANOVA  Sidak's multiple comparisons tests | P diet = 0.6384  P genotype = 0.0055  P interaction = 0.6426  Chow: P = 0.1537  HFD: P = 0.0477 |
| 4e | Two-way ANOVA  Sidak's multiple comparisons tests | P diet = 0.0440  P genotype = 0.1055  P interaction = 0.8594  Chow: P = 0.5033  HFD: P = 0.3728 |
| 4f | Two-way ANOVA  Sidak's multiple comparisons tests | P diet = 0.0115  P genotype = 0.1331  P interaction = 0.2478  Chow: P = 0.9591  HFD: P = 0.1297 |
| 4g | Two-way ANOVA  Sidak's multiple comparisons tests | P diet = 0.0075  P genotype = 0.3241  P interaction = 0.6473  Chow: P = 0.7830  HFD: P = 0.6231 |
| 4h | Two-way ANOVA  Sidak's multiple comparisons tests | P diet = 0.0142  P genotype = 0.4834  P interaction = 0.9740  Chow: P = 0.8515  HFD: P = 0.8580 |
| 4i | Two-way ANOVA  Sidak's multiple comparisons tests  Two-way ANOVA  Sidak's multiple comparisons tests | Females:  P diet = 0.0009  P genotype = 0.2715  P interaction = 0.1049  Chow: P = 0.9066  HFD: P = 0.1141  Males:  P diet = 0.0028  P genotype = 0.2446  P interaction = 0.2153  Chow: P = 0.9980  HFD: P = 0.1809 |
| 5a | One-way ANOVA  Tukey's multiple comparisons tests  One-way ANOVA  Tukey's multiple comparisons tests | Females:  P < 0.0001  Controls vs. *Igf2*^βKO^: P = 0.9646  Controls vs. *Lep*^ob/ob^: P < 0.0001  Controls vs. *Lep*^ob/ob^; *Igf2*^βKO^: P < 0.0001  *Igf2*^βKO^ vs. *Lep*^ob/ob^: P < 0.0001  *Igf2*^βKO^ vs. *Lep*^ob/ob^; *Igf2*^βKO^: P < 0.0001  *Lep*^ob/ob^ vs. *Lep*^ob/ob^; *Igf2*^βKO^: P = 0.9906  Males:  P < 0.0001  Controls vs. *Igf2*^βKO^: P > 0.9999  Controls vs. *Lep*^ob/ob^: P < 0.0001  Controls vs. *Lep*^ob/ob^; *Igf2*^βKO^: P < 0.0001  *Igf2*^βKO^ vs. *Lep*^ob/ob^: P < 0.0001  *Igf2*^βKO^ vs. *Lep*^ob/ob^; *Igf2*^βKO^: P < 0.0001  *Lep*^ob/ob^ vs. *Lep*^ob/ob^; *Igf2*^βKO^: P = 0.4013 |
| 5b | One-way ANOVA  Tukey's multiple comparisons tests  One-way ANOVA  Tukey's multiple comparisons tests | Females:  P < 0.0001  Controls vs. *Igf2*^βKO^: P = 0.9893  Controls vs. *Lep*^ob/ob^: P = 0.0031  Controls vs. *Lep*^ob/ob^; *Igf2*^βKO^: P < 0.0001  *Igf2*^βKO^ vs. *Lep*^ob/ob^: P = 0.0209  *Igf2*^βKO^ vs. *Lep*^ob/ob^; *Igf2*^βKO^: P < 0.0001  *Lep*^ob/ob^ vs. *Lep*^ob/ob^; *Igf2*^βKO^: P = 0.0017  Males:  P < 0.0001  Controls vs. *Igf2*^βKO^: P = 0.8089  Controls vs. *Lep*^ob/ob^: P = 0.0003  Controls vs. *Lep*^ob/ob^; *Igf2*^βKO^: P = 0.0003  *Igf2*^βKO^ vs. *Lep*^ob/ob^: P < 0.0001  *Igf2*^βKO^ vs. *Lep*^ob/ob^; *Igf2*^βKO^: P < 0.0001  *Lep*^ob/ob^ vs. *Lep*^ob/ob^; *Igf2*^βKO^: P = 0.9558 |
| 5c | One-way ANOVA  Tukey's multiple comparisons tests  One-way ANOVA  Tukey's multiple comparisons tests | Females:  P < 0.0001  Controls vs. *Igf2*^βKO^: P > 0.9999  Controls vs. *Lep*^ob/ob^: P = 0.0083  Controls vs. *Lep*^ob/ob^; *Igf2*^βKO^: P < 0.0001  *Igf2*^βKO^ vs. *Lep*^ob/ob^: P = 0.0268  *Igf2*^βKO^ vs. *Lep*^ob/ob^; *Igf2*^βKO^: P < 0.0001  *Lep*^ob/ob^ vs. *Lep*^ob/ob^; *Igf2*^βKO^: P = 0.2166  Males:  P = 0.0001  Controls vs. *Igf2*^βKO^: P = 0.8437  Controls vs. *Lep*^ob/ob^: P = 0.0111  Controls vs. *Lep*^ob/ob^; *Igf2*^βKO^: P = 0.0702  *Igf2*^βKO^ vs. *Lep*^ob/ob^: P = 0.0633  *Igf2*^βKO^ vs. *Lep*^ob/ob^; *Igf2*^βKO^: P = 0.2698  *Lep*^ob/ob^ vs. *Lep*^ob/ob^; *Igf2*^βKO^: P = 0.9410 |
| 5d | One-way ANOVA  Tukey's multiple comparisons tests  One-way ANOVA  Tukey's multiple comparisons tests | Females:  P = 0.0005  Controls vs. *Igf2*^βKO^: P = 0.9861  Controls vs. *Lep*^ob/ob^: P = 0.1390  Controls vs. *Lep*^ob/ob^; *Igf2*^βKO^: P = 0.0012  *Igf2*^βKO^ vs. *Lep*^ob/ob^: P = 0.1063  *Igf2*^βKO^ vs. *Lep*^ob/ob^; *Igf2*^βKO^: P = 0.0012  *Lep*^ob/ob^ vs. *Lep*^ob/ob^; *Igf2*^βKO^: P = 0.2179  Males:  P = 0.1368  Controls vs. *Igf2*^βKO^: P = 0.9768  Controls vs. *Lep*^ob/ob^: P = 0.1785  Controls vs. *Lep*^ob/ob^; *Igf2*^βKO^: P = 0.3944  *Igf2*^βKO^ vs. *Lep*^ob/ob^: P = 0.3169  *Igf2*^βKO^ vs. *Lep*^ob/ob^; *Igf2*^βKO^: P = 0.5973  *Lep*^ob/ob^ vs. *Lep*^ob/ob^; *Igf2*^βKO^: P = 0.9767 |
| S1d | Two-way ANOVA  Sidak's multiple comparisons tests  Two-way ANOVA  Sidak's multiple comparisons tests | *Ins2*:  P cell-type = 0.0059  P genotype = 0.7767  P interaction = 0.7960  β-cells (YFP^+^): P = 0.9158  Non β-cells (YFP^-^): P = 0.9998  *Amy2*:  P cell-type = 0.0447  P genotype = 0.5750  P interaction = 0.5753  β-cells (YFP^+^): P > 0.9999  Non β-cells (YFP^-^): P = 0.6758 |
| S2a | Two-way ANOVA  Sidak's multiple comparisons tests  Two-way ANOVA  Sidak's multiple comparisons tests  Two-way ANOVA  Sidak's multiple comparisons tests | Triglycerides:  P sex = 0.0003  P genotype = 0.6536  P interaction = 0.8267  Females: P = 0.9850  Males: P = 0.8527  Free fatty acids:  P sex = 0.0296  P genotype = 0.2990  P interaction = 0.7166  Females: P = 0.8673  Males: P = 0.5259  Cholesterol:  P sex < 0.0001  P genotype = 0.9327  P interaction = 0.6350  Females: P = 0.9573  Males: P = 0.8940 |
| S2b | Two-way ANOVA  Sidak's multiple comparisons tests | P sex = 0.0040  P genotype = 0.6635  P interaction = 0.5610  Females: P = 0.9943  Males: P = 0.6639 |
| S2c | Two-way ANOVA  Sidak's multiple comparisons tests  Two-way ANOVA  Sidak's multiple comparisons tests | Glucose:  P sex = 0.4800  P genotype = 0.0717  P interaction = 0.5402  Females: P = 0.6531  Males: P = 0.1379  Insulin:  P sex = 0.1228  P genotype = 0.8337  P interaction = 0.9010  Females: P = 0.9696  Males: P = 0.9973 |
| S3a | Two-way ANOVA  Sidak's multiple comparisons tests | P pregnancy < 0.0001  P genotype = 0.5704  P interaction = 0.8052  NP: P = 0.9996  E15: P = 0.9940  E19: P = 0.8242 |
| S3b | Two-way ANOVA  Sidak's multiple comparisons tests | P pregnancy = 0.0031  P genotype = 0.2197  P interaction = 0.5742  E15: P = 0.8712  E19: P = 0.3569 |
| S3c | Two-way ANOVA  Sidak's multiple comparisons tests | P pregnancy < 0.0001  P genotype = 0.3167  P interaction = 0.7113  NP: P > 0.9999  E15: P = 0.9233  E19: P = 0.6540 |
| S3d | One-way ANOVA  Tukey's multiple comparisons tests  One-way ANOVA  Tukey's multiple comparisons tests  One-way ANOVA  Tukey's multiple comparisons tests | Leptin:  P = 0.0097  C (NP) vs. C (P): P = 0.0134  C (NP) vs. *Igf2*^βKO^(P): P = 0.0257  C (P) vs. *Igf2*^βKO^(P): P = 0.9961  Adiponectin:  P = 0.0012  C (NP) vs. C (P): P = 0.0014  C (NP) vs. *Igf2*^βKO^(P): P = 0.0069  C (P) vs. *Igf2*^βKO^(P): P = 0.9535  Resistin:  P = 0.0004  C (NP) vs. C (P): P = 0.0008  C (NP) vs. *Igf2*^βKO^(P): P = 0.0012  C (P) vs. *Igf2*^βKO^(P): P = 0.9109 |
| S4a | Two-way ANOVA  Sidak's multiple comparisons tests  Two-way ANOVA  Sidak's multiple comparisons tests | Fat mass:  P age < 0.0001  P genotype = 0.1772  P interaction = 0.3580  8 weeks: P = 0.9413  28 weeks: P = 0.2101  Lean mass:  P age < 0.0001  P genotype = 0.9814  P interaction = 0.7500  8 weeks: P = 0.9632  28 weeks: P = 0.9729 |
| S4b | Two-way ANOVA  Sidak's multiple comparisons tests  Two-way ANOVA  Sidak's multiple comparisons tests | Fat mass:  P age < 0.0001  P genotype = 0.2083  P interaction = 0.5300  8 weeks: P = 0.8792  28 weeks: P = 0.3356  Lean mass:  P age < 0.0001  P genotype = 0.3046  P interaction = 0.8468  8 weeks: P = 0.6232  28 weeks: P = 0.8035 |
